# Therapeutic targeting of the eIF4E cap-binding domain reveals control of lineage fate in prostate cancer

**DOI:** 10.1172/JCI199838

**Published:** 2026-04-14

**Authors:** Rashmi Mishra, Sihyeon Song, Dhruv Choradia, Dmytro Rudoy, Cynthia L. Wladyka, Patrick Hoang, Jin Yeong Kim, Ilsa M. Coleman, Sonali Arora, Stephanie Dobersch, Alexander E. Orellana, Chenwei Lin, Philip R. Gafken, Eva Corey, Peter S. Nelson, Sita Kugel, Haolong Li, Arnab Sengupta, Andrew C. Hsieh

**Affiliations:** 1 Division of Human Biology, Fred Hutchinson Cancer Center, Seattle, Washington, USA.; 2 Department of Biology and; 3 Molecular and Cellular Biology Graduate Program, University of Washington, Seattle, Washington, USA.; 4 Proteomics & Metabolomics Shared Resource, Fred Hutchinson Cancer Center, Seattle, Washington, USA.; 5 Department of Urology, University of Washington, Seattle, Washington, USA.; 6 Division of Clinical Research, Fred Hutchinson Cancer Center, Seattle, Washington, USA.; 7 Departments of Medicine and Genome Sciences, University of Washington, Seattle, Washington, USA.; 8 Department of Biological and Environmental Sciences, Georgia College & State University, Milledgeville, Georgia, USA.

**Keywords:** Cell biology, Oncology, Molecular biology, Prostate cancer, Translation

## Abstract

Lineage plasticity underscores the resilience of cancer cells in the context of drug treatment. However, lineage fates can also be therapeutically directed. We demonstrate that the eukaryotic initiation factor 4E (eIF4E) cap-binding domain is a critical regulator of lineage plasticity in prostate cancer. Using a first-in-class cap-binding domain inhibitor, we found that plasticity is driven by translational repression of basal keratins through a shared cis-regulatory element enciphered in their 5’ untranslated regions (UTRs). Simultaneously, this stabilized the androgen receptor (AR) through translational upregulation of the deubiquitinases BAP1 and OTUD3. This lineage program is essential for cell survival and drives a druggable vulnerability. Notably, tumors resistant to AR blockade regained sensitivity upon eIF4E cap-binding domain inhibition, which reprogrammed them toward a luminal state. In patients with castration-resistant prostate cancer (CRPC), elevated eIF4E expression was associated with a basal phenotype, reduced luminal differentiation, and accelerated resistance to AR pathway inhibitors (ARPIs). These discoveries uncover a role for the eIF4E cap-binding domain in lineage plasticity and highlight that targeting this domain offers a promising strategy to overcome treatment resistance in prostate cancer.

## Abstract 2

Our study identifies a therapeutic approach for treatment-resistant prostate cancer by targeting the eIF4E cap-binding domain to drive basal-to-luminal lineage reprogramming via translational repression of basal keratins and stabilization of the AR protein.

## Graphical abstract

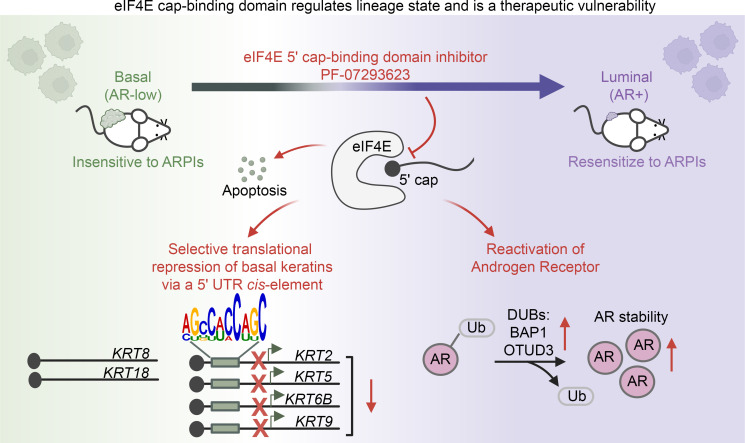

## Introduction

Prostate cancer is a leading cause of cancer-related mortality among men in the United States. It is estimated that in 2026, 333,830 men will be diagnosed with a prostate malignancy, and 36,320 men will perish from the disease (1). Despite advances in early detection and treatments, therapy resistance continues to drive poor outcomes, particularly in castration-resistant prostate cancer (CRPC). A common denominator of therapy resistance in CRPC is the transition between lineage states from androgen receptor–dependent (AR-dependent) to AR-independent phenotypes. These transitions may involve shifts to stem-like, progenitor, or multilineage intermediates, followed by either redifferentiation or transdifferentiation into alternative lineage identities (2). AR-driven prostate cancers are often classified as luminal subtypes, characterized by strong AR signaling, expression of luminal keratins (e.g., keratin 8 and 18), and responsiveness to AR pathway inhibitors (ARPI) (3, 4). In contrast, AR-independent tumors are typically classified as basal, exhibit limited AR signaling, express basal keratins (e.g., keratin 5, 6B, and 14), and are poorly responsive to ARPIs (4–6). Recent studies have broadened our understanding of lineage plasticity, documenting transition into diverse phenotypic states — including neuroendocrine, gastrointestinal, Wnt-dependent, stem-cell like, and club — and hillock-like states (7–11).

At a mechanistic level, these lineage states derive from distinct epigenetic and transcriptional programs (12–18). Lineage plasticity in cancer cells arises from signaling cascades that are triggered either due to mutational burden or as an escape mechanism to therapeutic pressure. Several key studies have uncovered resistance mechanisms of lineage switching in prostate cancer. For instance, resistance to enzalutamide has been linked to activation of the JAK-STAT pathway in TP53/RB1-deficient tumors via SOX2, promoting a shift toward a less luminal identity (12, 14, 19). Loss of PKCλ/ι fosters neuroendocrine prostate cancer (NEPC) features through EZH2-YY1 mediated transcriptional reprogramming, contributing to enzalutamide resistance (15). Additionally, AURKA and N-myc have been implicated in NEPC development, further emphasizing broad mechanisms that drive lineage plasticity in prostate cancer (7, 20).

Elucidating the mechanisms of lineage switching is crucial, as these transitions may be druggable and could be leveraged to resensitize prostate cancer to AR pathway inhibitors or prevent resistance altogether. Increasing evidence suggests that lineage plasticity in prostate cancer can be therapeutically targeted to restore sensitivity to AR inhibitors. For example, while ZNF397 deficiency can drive multilineage plasticity and ARPI resistance, this can be reversed with TET2 inhibitors (18). Notch signaling activation and ONECUT2 inhibition in NEPC can suppress neuroendocrine lineage plasticity to reduce growth and combat treatment resistance (16, 17). Similarly, inhibitors of JAK/STAT, FGFR, and EZH2 signaling pathways promote lineage switching to luminal subtypes and resensitization to ARPIs (13–15, 21–23). Together, these findings support the concept that targeting lineage plasticity is a viable strategy to overcome therapy resistance in prostate cancer.

While substantial progress has been made in characterizing the epigenetic and transcriptional drivers of lineage states in CRPC, it remains unknown if and how mRNA translation regulation plays a role in this process. This is an important question because components of the translation initiation complex, including eukaryotic initiation factor 4E (eIF4E), eukaryotic initiation factor 3B (eIF3B), and eIF4E binding protein 1 (4EBP1), have been mechanistically implicated in promoting AR independence and castration resistance (24–27). In this study, we identify the eIF4E cap-binding domain as a critical gatekeeper of lineage state in prostate cancer. Therapeutic targeting of this domain using a small molecule that sequesters the 7-methylguanosine cap-binding site induces a basal-to-luminal lineage switch. This is mediated through sequence-specific repression of basal keratin mRNA translation and deubiquitinase regulation of AR protein stability. In samples from patients with metastatic CRPC, elevated eIF4E expression correlates with a basal phenotype and rapid onset of ARPI resistance. To determine if this is clinically relevant, we conducted preclinical trials and found that eIF4E cap-binding domain inhibition restores sensitivity to ARPI therapy by reprogramming basal tumors to a luminal phenotype. Collectively, these findings uncover an unexpected role for mRNA-specific translation in governing lineage plasticity and the therapeutic response in prostate cancer.

## Results

### The eIF4E cap-binding domain is a new therapeutic vulnerability in advanced prostate cancer.

**Figure 1:**
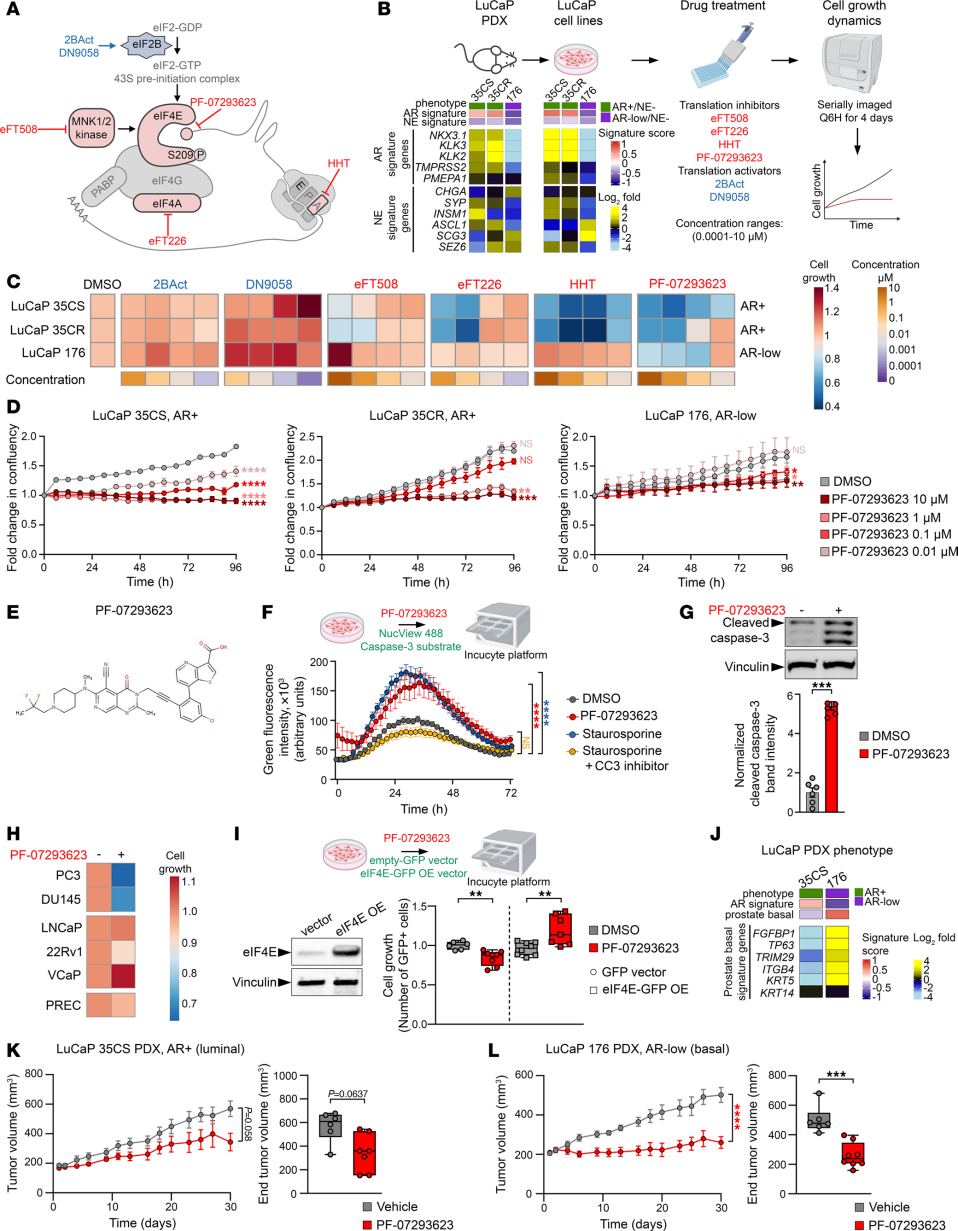
Inhibition of the eIF4E cap-binding domain suppresses advanced prostate cancer growth. (**A**) Schematic showing mechanism of action of clinical and preclinical mRNA translation modulators: 2BAct, DN9058, eFT508, eFT226, HHT, and PF-07293623. (**B**) Preclinical pharmacological screen on LuCaP cell lines. (**C**) Heatmap showing fold change in growth of LuCaP cells treated with DMSO (vehicle) or translation activators or inhibitors. *n* = 3 or more. (**D**) Cell growth curves of LuCaP 35CS, LuCaP 35CR, and LuCaP 176 cells treated with DMSO or PF-07293623, normalized to vehicle. (**E**) Chemical structure of PF-07293623. (**F**) Caspase-3 activity over time in LuCaP 176 cells treated with DMSO or PF-07293623 (100 nM) treatment. *n* = 4. In controls, Staurosporin was used to induce apoptosis and C3 inhibitor was used to inhibit apoptosis. (**G**) Representative immunoblots (top) and quantification (bottom) of cleaved caspase 3 in LuCaP 176 cells treated with DMSO or PF07293623 (100 nM, 72 hours), *n* = 6. (**H**) Heatmap showing fold change in growth of PC3, DU145, LNCaP, 22Rv1, VCaP, and PREC cells treated with DMSO or PF-07293623 (100 nM), *n* = 3. (**I**) Cell growth of GFP+ cells expressing vector or eIF4E-GFP constructs treated with DMSO or PF-07293623 (100 nM, 72 hours). (**J**) Luminal and basal features of LuCaP PDX models. (**K** and **L**) LuCaP PDX tumor growth curve (left) and end tumor volumes (right) for (**K**), LuCaP 35CS PDX treated with vehicle (*n* = 6), PF07293623 (*n* = 7), and (**L**) LuCaP 176 PDX treated with vehicle (*n* = 6) or PF07293623 (*n* = 8). Plots represent mean ± SEM. Significance was determined by 1-way ANOVA with Dunnett’s multiple comparisons test in **D**, **F**; by Unpaired 2-tailed Student’s *t* test in **G**, **I**, **K**, and **L**. *P < 0.05; **P < 0.01; ***P < 0.001; ****P < 0.0001.

Previous studies of translation inhibitors and activators in cancer have primarily focused on the central premise that suppressing or enhancing protein synthesis directly yields antitumor effects. However, no systematic comparison of these agents has been made across the same models in a human malignancy. This raises the important question of the comparative efficacy of each modality. We conducted a pharmacological screen of translation modulators, both inhibitors and activators, probing their sensitivity across a panel of advanced-stage prostate cancers. We tested a series of preclinical and clinical compounds that either enhance or inhibit protein synthesis, including 2BAct (eIF2B activator), DN9058 (eIF2B activator, Denali Therapeutics), eFT508 (eIF4E serine 209 phosphorylation inhibitor, eFFECTOR Therapeutics), eFT226 (eIF4A inhibitor, eFFECTOR Therapeutics), homoharringtonine (HHT) (translation initiation elongation inhibitor, Teva), and PF-07293623 (eIF4E cap-binding domain inhibitor, Pfizer) (Figure 1A) (28–32). We screened each compound across a panel of CRPC patient–derived xenograft (PDX) models that have been adapted to 2D culture: LuCaP 35CS (AR-intact castration sensitive in vivo), LuCaP 35CR (AR-intact castration resistant in vivo), and LuCaP 176 (AR-low CRPC) (Figure 1B) (33). These models were chosen because they represent major phenotypes observed in patients with prostate cancer. Importantly, transcriptional profiling revealed strong similarities between the 2D and PDX models (Figure 1B).

The compounds were screened for effects on cell growth by serial imaging over 4 days at a concentration range of 0.0001–10 μM. Unexpectedly, we found that the eIF2B activators 2BAct and DN9058 did not inhibit the growth of any LuCaP model (Figure 1C and Supplemental Figure 1, A and B; supplemental material available online with this article; https://doi.org/10.1172/JCI199838DS1). Among the translation inhibitors, eFT508 exerted a slight reduction in growth in AR-proficient LuCaP 35CS and 35CR lines, though only at higher doses (Figure 1C and Supplemental Figure 1C). However, eFT226 and HHT more effectively suppressed the growth of these same models at lower doses (Figure 1C and Supplemental Figure 1, D and E). Notably, none of these agents had any significant impact on the growth of AR-low LuCaP 176 cells (Figure 1C). In contrast, PF-07293623 consistently inhibited growth across all 3 LuCaP models (Figure 1, C and D). PF-07293623 is a 7-(5-chloro-2-{3-(5-cyano-6-{(1-(2,2-difluoropropyl)piperidin-4-yl) (methyl)amino}-2-methyl-4-oxopyrido[3,4-d]pyrimidin-3(4H)-yl)prop-1-yn-1-yl}phenyl)thieno[3,2-b]pyridine-3-carboxylic acid that was identified through a medicinal chemistry campaign to directly inhibit the eIF4E cap-binding domain (Figure 1E) (32). We next sought to determine the cellular mechanism through which PF-07293623 disrupts cell growth. We observed a small but significant increase in the G1 phase, accompanied by a reduction in the S and G2/M phases of the cell cycle (Supplemental Figure 1F). Additionally, utilizing real-time cleaved caspase 3 monitoring in live cells, we observed a substantial increase in apoptosis upon drug treatment, which was also confirmed by Western blot analysis (Figure 1, F and G). Given the profound effects of the agent on AR-low prostate cancer growth, we next tested the drug in AR-null PC3 and DU145 cells and compared the response to AR-driven LNCaP, 22Rv1, and VCaP cells. Strikingly, the AR-null cells were highly sensitive to cap-binding domain inhibition, whereas the AR-driven cells were largely resistant, suggesting that dependence on AR signaling may limit vulnerability (Figure 1H and Supplemental Figure 2, A–E). Since eIF4E cap-binding domain inhibition could theoretically be broadly toxic, we tested this compound in immortalized PrEC cells and found that they were insensitive to the drug (Figure 1H and Supplemental Figure 2F). These data show that inhibition of the eIF4E cap-binding domain by PF-07293623 can target AR-independent cells. To further evaluate if eIF4E directly mediates this sensitivity, we overexpressed eIF4E in LuCaP 176 cells and assessed the impact of PF-07293623 on cell growth. Overexpression of eIF4E rescued the drug-induced growth inhibition observed in vector controls (Figure 1I). In addition, eIF4E overexpression led to an increase in cell size, consistent with a conserved role of eIF4E in promoting cell growth (Supplemental Figure 2G) (34).

These findings led us to investigate if targeting the eIF4E cap-binding domain could effectively suppress tumor growth in vivo without undue toxicity. We chose to test the CRPC LuCaP 35CS PDX model, which expresses AR and is luminal in nature, and the LuCaP 176 PDX model, which has basal features and expresses low levels of AR similar to their in vitro counter parts (Figure 1, B and J, and Supplemental Figure 2H). Importantly, basal prostate cancers are more aggressive and resistant to therapies compared with luminal prostate cancers (35). Mice were randomized to receive vehicle or PF-07293623 (50 mg/kg, orally, twice daily) (Supplemental Figure 2I). The luminal LuCaP 35CS model exhibited a modest response to PF-07293623 treatment (Figure 1K). In contrast, the basal LuCaP 176 model was remarkably responsive to eIF4E cap-binding domain inhibition. In fact, tumors remained the same size throughout the trial, revealing a complete block in growth (Figure 1L). Importantly, mice treated with PF-07293623 showed no signs of significant toxicity (Supplemental Figure 2, J–N). Together, these findings demonstrate the therapeutic efficacy and safety of targeting the eIF4E cap-binding domain in advanced prostate cancer. Moreover, the agent appears to be particularly effective in recalcitrant prostate cancer with basal features.

### PF-07293623 inhibits protein synthesis and cap binding to eIF4E.

**Figure 2:**
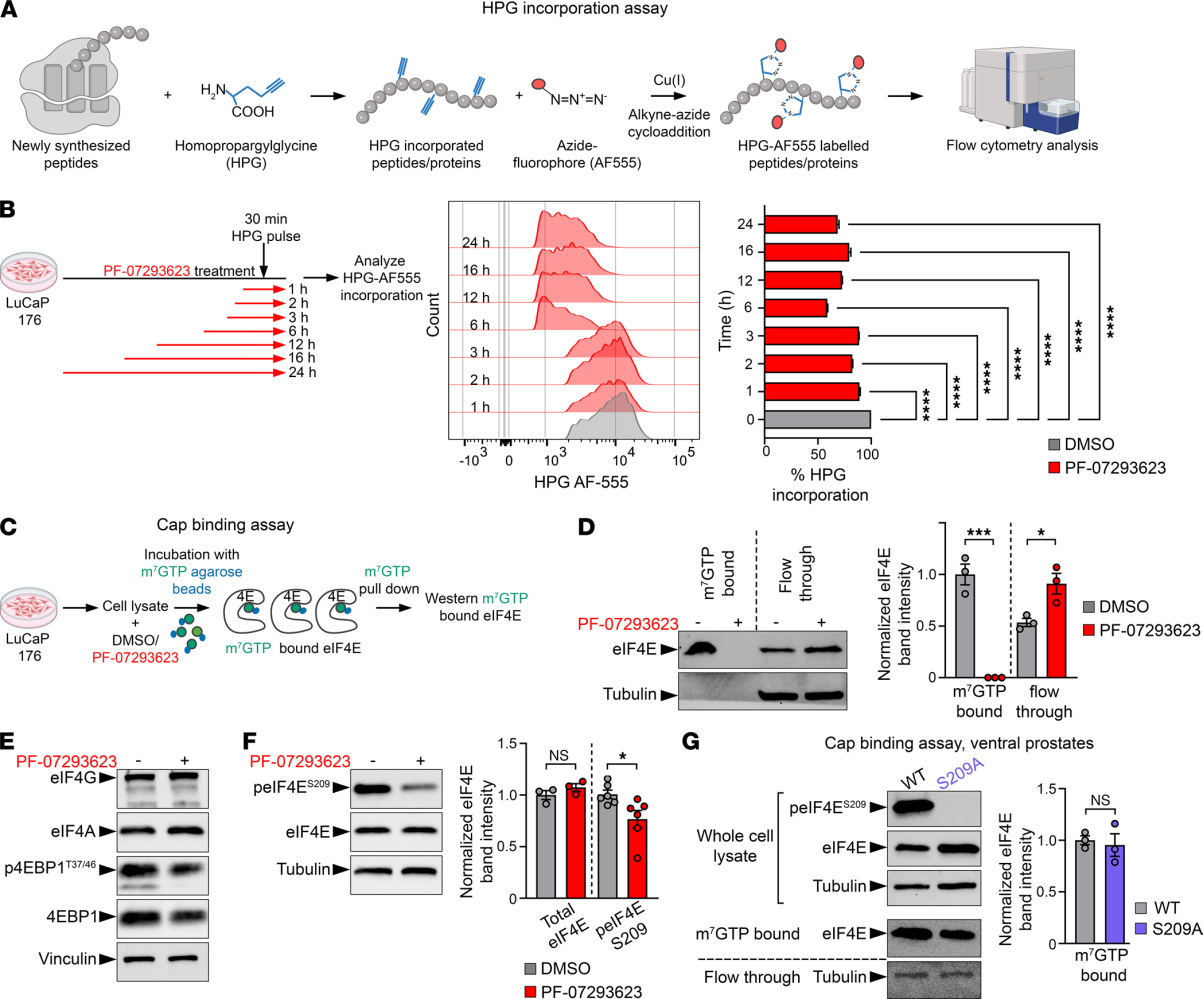
PF-07293623 impairs protein synthesis rates by suppressing eIF4E-m^7^G cap-binding affinity. (**A**) Schematic of the homopropargylglycine (HPG) incorporation assay. HPG, a methionine analog, is incorporated into newly synthesized proteins and conjugated to AF555 via copper-catalyzed click chemistry; fluorescence measured by flow cytometry reflects protein synthesis. (**B**) Representative HPG incorporation histograms (middle) and fluorescence quantification (right) in LuCaP 176 cells treated with DMSO (vehicle) or PF-07293623 (100 nM). (**C**) Schematic of m^7^G cap-binding assay. (**D**) Representative immunoblot (left) and quantification (right) of eIF4E in m^7^G bound and flow-through fractions in LuCaP 176 cells treated with DMSO or PF-07293623 (100 nM, 6 hours), *n* = 3. (**E** and **F**) Representative immunoblots of LuCaP 176 cells treated with DMSO or PF-07293623 (100 nM, 72 hours), *n* = 3 or more for (**E**) eIF4G, eIF4A, phospho-4EBP1^T37/46^, and 4EBP1, and (**F**) phospho-eIF4E^S209^ and eIF4E. (**G**) Representative immunoblot and quantification of phospho-eIF4E (S209) and eIF4E in whole cell lysates (top) and m^7^G bound fractions (bottom) from ventral prostates of WT and eIF4E S209A mice, *n* = 3 per group. Plots represent mean ± SEM. Significance was determined by 1-way ANOVA with Dunnett’s multiple comparisons test in **B**; by Unpaired 2-tailed Student’s *t* test in **D**, **F**, and **G**. *P < 0.05; ***P < 0.001; ****P < 0.0001.

eIF4E binds to the 7-methylguanosine (m^7^G) cap of mRNA to initiate mRNA translation (36). PF-07293623 was developed as an eIF4E cap-binding domain inhibitor and should therefore inhibit protein synthesis. To monitor the effects PF-07293623 on mRNA translation, we employed biorthogonal noncanonical amino acid tagging (BONCAT) with homopropargylglycine (HPG), a methionine analog with an alkyne moiety that incorporates into nascent peptide chains in place of endogenous methionine (37). HPG-labeled peptides can be selectively tagged with a fluorophore via a copper-catalyzed azide-alkyne cycloaddition reaction, enabling quantification of newly synthesized proteins within cells by flow cytometry (Figure 2A). We measured HPG incorporation after PF-07293623 treatment over a 24-hour period and observed a decrease in protein synthesis after 2 hours of drug exposure, which peaked by 6 hours (Figure 2B). Overall, PF-07293623 decreased mRNA translation by 40% (Figure 2B).

The rationale behind eIF4E cap-binding domain inhibitors is to compete against capped mRNA for binding to eIF4E, thereby inhibiting cap-dependent mRNA translation (38). We sought to directly verify whether PF-07293623 acts as a cap mimetic. To test this, we pretreated LuCaP 176 cells with or without PF-07293623 and performed a cap-binding assay to assess the affinity of eIF4E for a m^7^G cap analog which mimics capped mRNA (Figure 2C). PF-07293623 treatment strikingly blocked binding of eIF4E to the m^7^G cap analog and increased the levels of unbound eIF4E in the flow-through fraction (Figure 2D). Importantly, PF-07293623 did not affect total levels of eIF4E or coassociated proteins such as eIF4G, eIF4A, and 4EBP1 (Figure 2, E and F, and Supplemental Figure 3A).

Interestingly, PF-07293623 treatment led to a decrease in eIF4E phosphorylation at serine 209 (Figure 2F). In vitro studies with purified eIF4E have presented contradictory findings of the role of serine 209 phosphorylation on cap-binding capacity (39, 40). As such, we sought to determine how eIF4E serine 209 phosphorylation impacts cap binding in prostate epithelial cells. To this end, we performed cap-binding assays of prostate epithelial cells isolated from eIF4E knock-in mice where serine 209 has been mutated to an alanine and is therefore unphosphorylatable (Figure 2G and Supplemental Figure 3B) (41). Remarkably, we found no difference in the binding affinity of WT and mutant eIF4E (S209A) to the m^7^G cap analog (Figure 2G). This finding suggests that PF-07293623 cap-binding inhibition likely functions independent of phosphorylation of eIF4E at serine 209. Together, this data shows that PF-07293623 is a mRNA translation inhibitor, which functions by disrupting eIF4E binding to the m^7^G cap.

### The eIF4E cap-binding domain controls lineage state.

**Figure 3:**
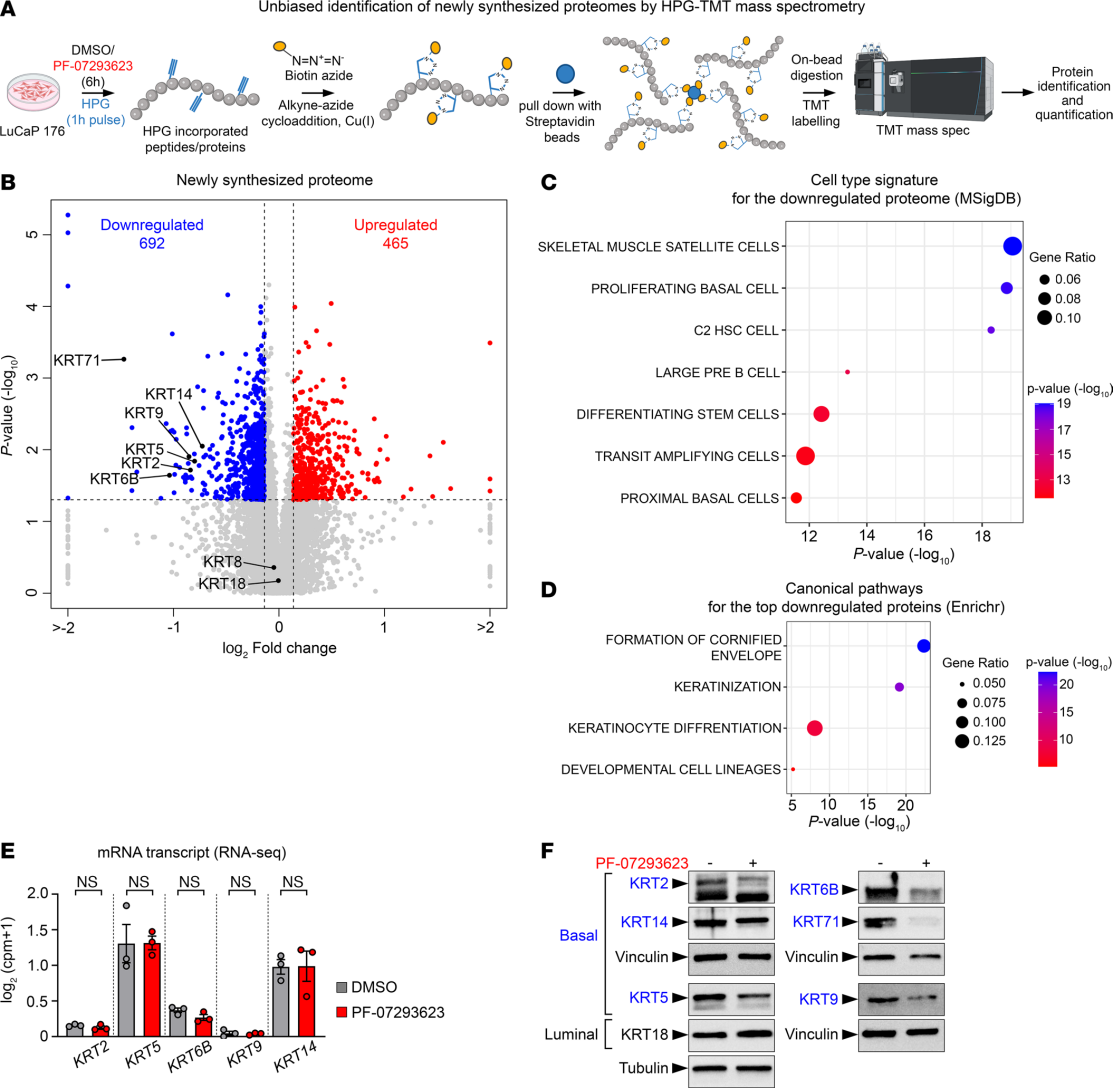
Loss of basal cell identity upon inhibition of the eIF4E cap-binding domain. (**A**) Schematic of HPG-TMT mass spectrometry. (**B**) Volcano plot of differentially regulated proteins in LuCaP 176 cells upon eIF4E cap-binding domain inhibition with PF-07293623 (100 nM, 6 hours). (**C**) GSEA of cell type signature (C8, MSigDB) of top 500 downregulated proteins shown in **B**. (**D**) GSEA of canonical pathways (CP, Enrichr) for top 50 downregulated proteins shown in **B**. (**E**) mRNA levels of *KRT2*, *KRT5*, *KRT6B*, *KRT9,* and *KRT14*. (**F**) Representative immunoblots for KRT2, KRT5, KRT6B, KRT9, KRT14, KRT71, and KRT18 in LuCaP 176 cells treated with PF-07293623 (100 nM, 72 hours), normalized to vehicle, *n* = 3. Plots represent mean ± SEM. Significance was determined by Unpaired 2-tailed Student’s *t* test in **E**.

To elucidate the mechanism by which inhibiting the cap-binding domain of eIF4E suppresses prostate cancer growth in the setting of low AR, we sought to quantify the therapeutic proteome upon drug treatment. However, conventional mass spectrometry can only measure steady-state levels of proteins and may be influenced by turnover. To selectively quantify newly synthesized proteins upon cap-binding domain inhibition, we employed a state-of-the-art approach that combines HPG labeling with mass spectrometry. Unlike conventional proteomics, HPG-TMT mass spectrometry specifically captures newly synthesized proteins through HPG pulse labeling and pulldown (Figure 3A). This approach provides a more accurate and time-resolved snapshot of proteome-wide translational activity.

AR-low LuCaP 176 cells were treated with PF-07293623 or vehicle for 6 hours, which correlates to the peak of protein synthesis inhibition (Figure 2B), and subjected to HPG-TMT mass spectrometry (Figure 3A). PF-07293623 treatment resulted in 692 downregulated and 465 upregulated proteins (Figure 3B and Supplemental Table 1). Interestingly, Gene Set Enrichment Analysis (GSEA) of the top 500 downregulated proteins revealed enrichment of basal-cell type gene signatures (Figure 3C). In parallel, GSEA of the most strongly downregulated proteins (top 50 candidates) identified significant enrichment of keratinization-related canonical pathways (CP) and gene ontology (GO) terms (Figure 3D and Supplemental Figure 4A). Collectively, we found that these signatures were associated with basal keratins (KRT), including KRT 2, 5, 6B, and 14, as well as KRT 9 and 71 (Figure 3B). RNA-seq and Western blot analysis revealed that the expression of these basal keratins was not reduced at the mRNA level but was decreased at the protein level following treatment with PF-07293623 (Figure 3, E and F, Supplemental Figure 4B, and Supplemental Table 2). Importantly, we observed no changes in luminal KRT 8 and 18, suggesting that eIF4E cap-binding domain inhibition specifically affects the translation of basal keratins (Figure 3, B and F). These results suggest that the eIF4E cap-binding domain can control lineage features of prostate cancer through the selective translation of basal keratins.

### Basal keratins are regulated by a 5′ UTR cis-regulatory element and are necessary for survival.

**Figure 4:**
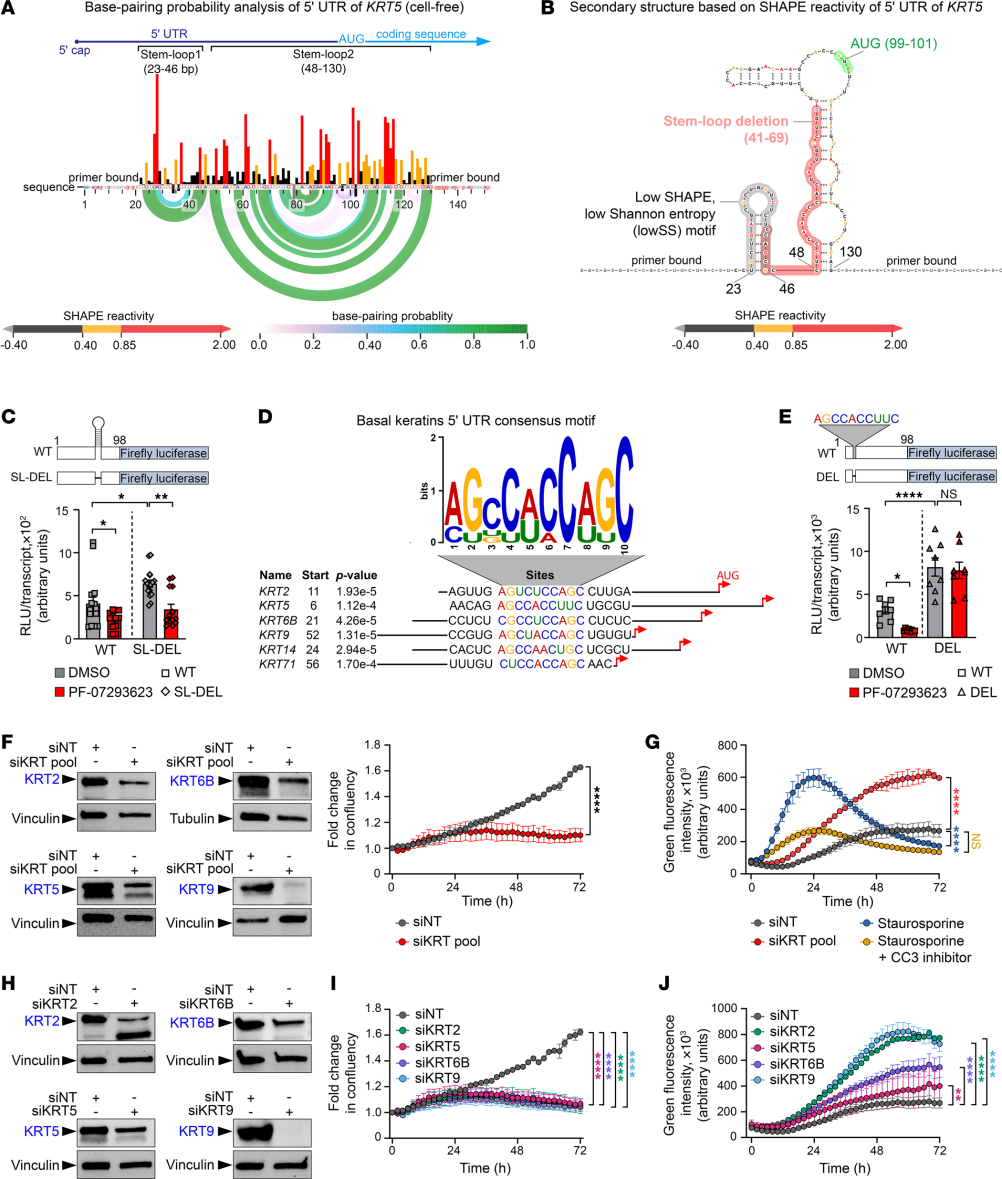
Basal keratins are translationally controlled and required for cell survival. (**A** and **B**) Cell-free SHAPE reactivity of the 5’ UTR of *KRT5* from LuCaP 176 cells showing (**A**) base pairing probability and (**B**) Secondary structure. (**C**) KRT5 5′ UTR luciferase assay with stem-loop mutant (Δ41–69), normalized to luciferase mRNA, *n* = 11 or more. (**D**) Consensus motif identified within basal keratin 5’ UTRs by MEME Stream. (**E**) *KRT5* 5′ UTR luciferase assay with motif mutant (Δ6–15), normalized to luciferase mRNA, *n* = 8 or more. (**F** and **G**) Pooled keratin (siKRT pool) or nontarget control (siNT) knockdown, *n* = 3, showing (**F**), keratin protein expression (left) and growth curves (right), as well as (**G**) caspase-3 activity over time. (**H**–**J**) Individual keratin knockdown (siKRTs), *n* = 3, showing (**H**) keratin protein expression (**I**), growth curves, and (**J**) caspase-3 activity over time. Plots represent mean ± SEM. Significance was determined by 1-way ANOVA with Šidák’s multiple comparisons test in **C**, **E**; by one way ANOVA with Dunnett’s multiple comparisons test in **G**, **I**, and **J**; by Unpaired 2-tailed Student’s *t* test in **F**. *P < 0.05; **P < 0.01; ****P < 0.0001.

To determine the mechanism by which the eIF4E cap-binding domain coordinates the translation of basal keratins, we focused on the 5′ untranslated region (5′ UTR). The 5′ UTR is situated upstream of the coding sequence of nearly all mRNAs and its capped 5′ end is the docking site for eIF4E (42). We hypothesized that specific sequence motifs or structure-based elements within the 5′ UTR of basal keratin mRNAs influence their translational efficiency and sensitivity to PF-07293623. Building on this concept, we investigated the 5′ UTR of *KRT5*, a canonical basal marker, to identify cell-type–specific structural features that might confer translational control. We performed selective 2′-hydroxyl acylation analyzed by primer extension and mutational profiling (SHAPE-MaP) using *KRT5* gene-specific reverse transcription on both gently deproteinated cell-free RNA and in-cell RNA from LuCaP 176 cells. We identified 2 stem-loop structures within the *KRT5* 5′ UTR that showed high base-pairing probability in both cell-free and in-cell conditions (Figure 4, A and B, and Supplemental Figure 5A). The first, spanning nucleotides 23–46, exhibited low SHAPE reactivity (< 0.4) and low Shannon entropy (< 0.08), suggestive of a stable and potentially functional structural element (43). The second region, spanning nucleotides 48–130 expands into the coding sequence, which starts at position 99. This structure had higher SHAPE and Shannon entropy (> 0.4 and > 0.08, respectively) making it more dynamic and less stable (Figure 4, A and B). To determine whether these structures contribute to translation regulation, we generated luciferase reporter constructs in which nucleotides 41–69 were deleted to disrupt both stem loops simultaneously (Figure 4B). WT and the stem-loop deletion mutant constructs were transduced in LuCaP 176 cells and treated with PF-07293623. We observed that deletion of the stem loop increased translation through the *KRT5* 5′ UTR (Figure 4C). However, both the WT and mutant 5′ UTR remained sensitive to eIF4E cap-binding domain inhibition (Figure 4C). These results suggest that the stem loops we identified are not responsible for the translation control of *KRT5* upon treatment with PF-07293623.

Given these findings, we next examined the 5′ UTR sequences of *KRT*2, *5*, *6B*, *9*, *14*, and *71* to identify regulatory motifs that could potentially mediate translational responsiveness to PF-07293623. Using MEME STREAM analysis, we identified a conserved 10 base cis-regulatory element, AGCCWCCAGC, selectively present in the 5′ UTRs of these keratins but absent in the 5′ UTRs of luminal keratins *KRT8* and *KRT18*, suggesting basal lineage specificity (Figure 4D). This motif was also enriched in the 5′ UTRs of 42 of the top 50 transcripts downregulated upon cap-binding domain inhibition, with a total of 215 motif occurrences (Supplemental Figure 5B and Supplemental Table 3), suggesting a potential role in mediating translational sensitivity. To assess the functional significance of this cis-element, we generated a luciferase reporter construct harboring a 10-base deletion (positions 6–15) within the *KRT5* 5′ UTR, which we transduced into LuCaP 176 cells. This deletion resulted in a significant increase in luciferase activity, indicating that the motif acts as a translational repressor (Figure 4E). Furthermore, while the WT *KRT5* 5′ UTR was sensitive to PF-07293623, the mutant lacking the cis-element was unresponsive to PF-07293623 treatment, suggesting that the cis-regulatory motif is required for PF-07293623–mediated translational repression (Figure 4E). Together, these findings demonstrate that inhibition of the eIF4E cap-binding domain in AR-low CRPC selectively suppresses the translation of basal keratins, contributing to lineage plasticity through a conserved 10-base cis-regulatory element located within their 5′ UTRs.

While it is known that keratins can define lineage states, it is unknown if basal keratins themselves are required for survival of prostate cancer. This is an important question because basal keratins are functional targets of PF-07293623. To this end, we conducted a pooled knockdown of KRT 2, 5, 6B, and 9 and observed a significant decrease in cellular growth capacity, which was accompanied by a substantial increase in apoptosis (Figure 4, F and G). Next, we conducted individual knockdown of each of these basal keratins and found they were all essential for cell growth and survival (Figure 4, H–J). Thus, in AR-low prostate cancer, keratins are not only markers of the basal phenotype but required for cell survival.

### Inhibition of the eIF4E cap binding induces lineage plasticity through BAP1 and OTUD3.

**Figure 5:**
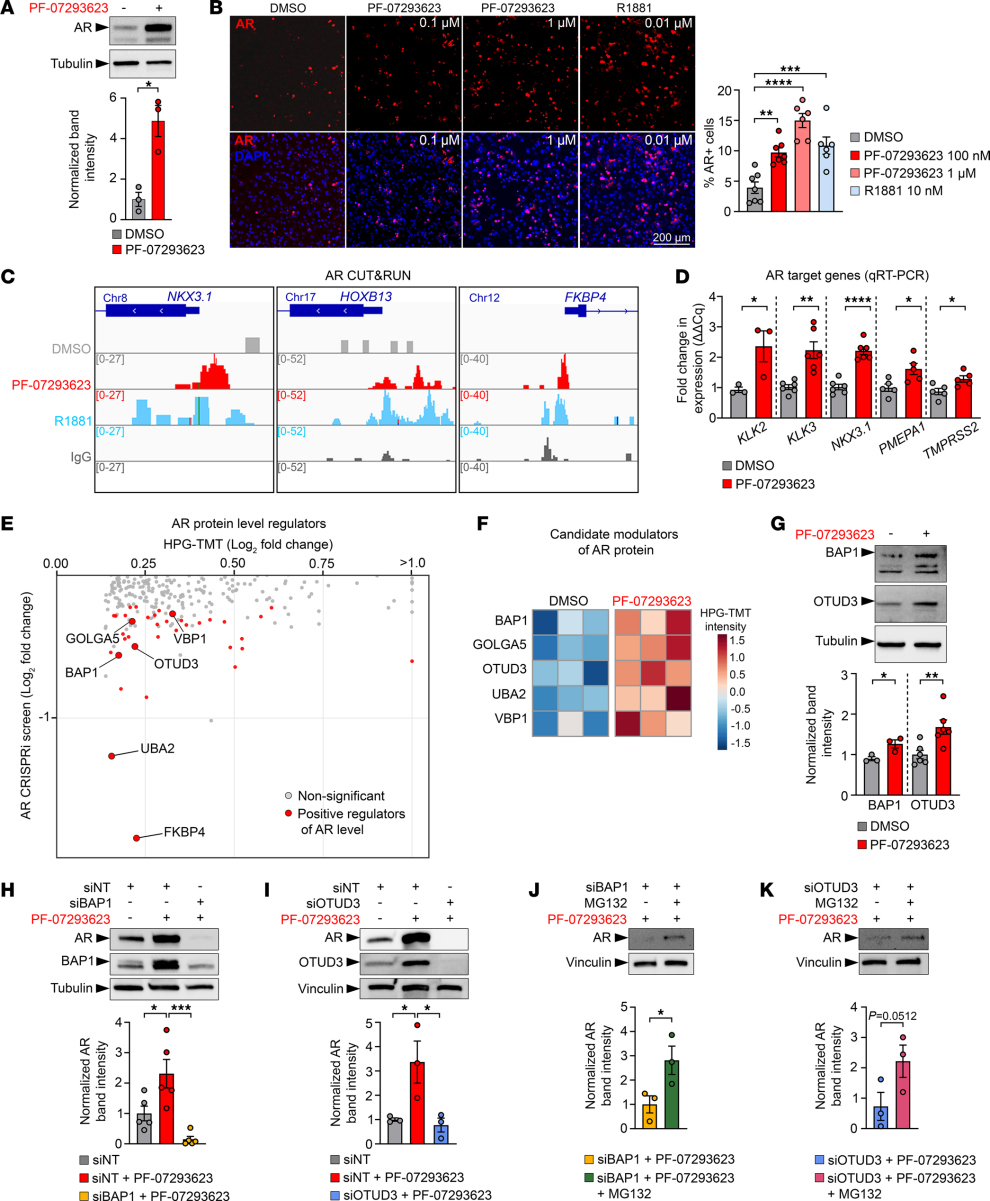
Deubiquitinases OTUD3 and BAP1 prevent AR degradation upon eIF4E cap-binding domain inhibition. (**A**) Immunoblot of AR in LuCaP 176 cells treated with DMSO (vehicle) or PF07293623 (100 nM, 72 hours), *n* = 3. (**B**) AR Immunofluorescence images (left) and quantification (right) in LuCaP 176 cells treated with DMSO or PF07293623 (100 nM, 1 μM) or R1881 (10 nM), *n* = 6 or more. Scale bar: 200 μm. (**C**) Representative CUT&RUN peaks showing AR binding, *n* = 3. (**D**) qRT-PCR analysis of AR target genes in LuCaP 176 cells. (**E**) Scatter plot of candidates from HPGTMT mass spectrometry and AR CRISPRi screen. Significant overlapping positive AR regulators shown in red. (**F**) Heatmap showing HPG-TMT expression intensity of candidate AR regulators. (**G**) BAP1 and OTUD3 immunoblots upon PF07293623 treatment (100 nM). (**H**–**K**) Representative immunoblot (top) and quantification (bottom) of AR in LuCaP 176 cells treated with PF07293623 (100 nM) and transfected with nontargeting siRNA (siNT) or (**H**) siBAP1, (**I**) siOTUD3, (**J**) siBAP1 and MG132 (10 μM, 6 hours), (**K**) siOTUD3 and MG132. Plots represent mean ± SEM. Significance was determined by unpaired 2-tailed Student’s *t* test in **A**, **D**, **G**, **J**, and **K,** *n* = 3; by 1-way ANOVA with Dunnett’s multiple comparisons test in **B**; by 1-way ANOVA with Holm-Šidák’s multiple comparisons test in **H** and **I**, *n* = 3. *P < 0.05; **P < 0.01; ***P < 0.001; ****P < 0.0001.

Since inhibition of the eIF4E cap-binding domain reduces the basal phenotype in AR-low prostate cancer (Figure 3, B and F), we next asked whether this shift is accompanied by a corresponding gain in luminal identity. Given that AR expression is a defining marker of luminal prostate cells, we examined AR protein levels following treatment of LuCaP 176 cells with PF-07293623. Remarkably, inhibition of the eIF4E cap-binding domain led to a 5-fold increase in AR expression (Figure 5A). This increase was dose dependent and primarily observed in the nucleus, where AR functions as a transcription factor (Figure 5B and Supplemental Figure 6A). This finding indicates that inhibition of the eIF4E cap-binding domain drives aggressive basal prostate cancer cells toward a more luminal state. To determine whether the observed increase in AR protein was due to elevated AR transcript levels, we examined AR mRNA expression. Notably, the increase in AR protein did not correspond to any change in AR transcript levels, suggesting a posttranscriptional mechanism of regulation (Supplemental Figure 6B and Supplemental Table 2). Next, we analyzed protein synthesis rates of AR via HPG-TMT mass spectrometry. Consistent with AR mRNA expression, there was no difference in the levels of newly synthesized AR (Supplemental Figure 6C). At a functional level, PF-07293623 treatment enhanced AR chromatin occupancy at its canonical targets. AR CUT&RUN demonstrated increased AR binding at the transcription start sites of *NKX3.1*, *HOXB13*, and *FKBP4*, and ChIP-qPCR revealed increased AR occupancy at *FKBP5*, *KLK3*, and *TMPRSS2* genomic loci (Figure 5C and Supplemental Figure 6D). Increased AR chromatin binding was accompanied by higher expression of *KLK2*, *KLK3*, *NKX3.1*, *PMEPA1*, and *TMPRSS2* transcripts (Figure 5D). These findings suggest that PF-07293623 increases AR protein levels and initiates active AR signaling through a mechanism independent of transcriptional or translational control.

Given these findings, we performed canonical pathway enrichment analysis on the newly synthesized proteome sensitive to PF-07293623 (Figure 3B). Interestingly, posttranslational protein modification emerged as one of the most significantly enriched pathways in the upregulated but not the downregulated proteins (Supplemental Figure 6, E and F). To further narrow down functional posttranslational regulators of AR, we compared our upregulated proteins to a genome-scale CRISPRi screen for positive regulators of AR expression (Supplemental Figure 6G) (44). Analyzing the intersection of these datasets, we identified 36 proteins that, when inhibited by CRISPRi decreased AR levels suggesting they are necessary for AR expression (log_2_ fold change > 0.2 or < –0.2; *P* < 0.05) (Figure 5E and Supplemental Figure 6H). Notably, one of the top overlapping hits was FKBP4, a well-characterized regulator of AR stability and function supporting the robustness of this analysis (Figure 5E) (45). These findings suggest that a subset of upregulated proteins may be responsible for the increase in AR abundance upon eIF4E cap-binding domain inhibition.

At a mechanistic level, we focused on candidates that are known posttranslational modifiers and associated with prostate cancer or AR biology including, BRCA1-associated protein (BAP1), golgin subfamily A member 5 (GOLGA5), ovarian tumor domain–containing protein 3 (OTUD3), ubiquitin-like modifier activating enzyme 2 (UBA2), and von Hippel-Lindau-binding protein 1 (VBP1) (Figure 5F). BAP1 has been shown to suppress prostate cancer growth through deubiquitinating PTEN (46). GOLGA5 is an endosomal and vesicular transport protein that binds to the N-terminus of AR (47). OTUD3 deubiquitinates PTEN and YY1, both of which directly interact with AR (48, 49); UBA2 is an E1 SUMO-1 activating enzyme that conjugates with UBC9 to SUMOylate AR (50, 51). VBP1 is a component of the VHL-Elongin B/C E3 ligase complex that also deubiquitinates AR (52). To validate if these proteins are upregulated in the context of PF-07293623 treatment, we conducted Western blot analysis. Among this group, the steady-state protein levels of 2 candidates, BAP1 and OTUD3, increased upon cap-binding domain inhibition without any changes at the mRNA level (Figure 5G and Supplemental Figure 6, I and J). Both proteins are deubiquitinases, but it is unknown if they are necessary for AR protein stability. To this end, we knocked down BAP1 or OTUD3 with and without eIF4E cap-binding domain inhibition. We found that knockdown of BAP1 or OTUD3 prevented the up regulation of AR in response to PF-07293623 treatment (Figure 5, H and I), which could be rescued by the proteasome inhibitor MG-132 (Figure 5, J and K). These findings demonstrate that BAP1 and OTUD3 contribute to AR protein stabilization in response to eIF4E cap-binding domain inhibition.

### eIF4E expression is associated with a basal phenotype and worse outcomes in patients with advanced prostate cancer.

**Figure 6:**
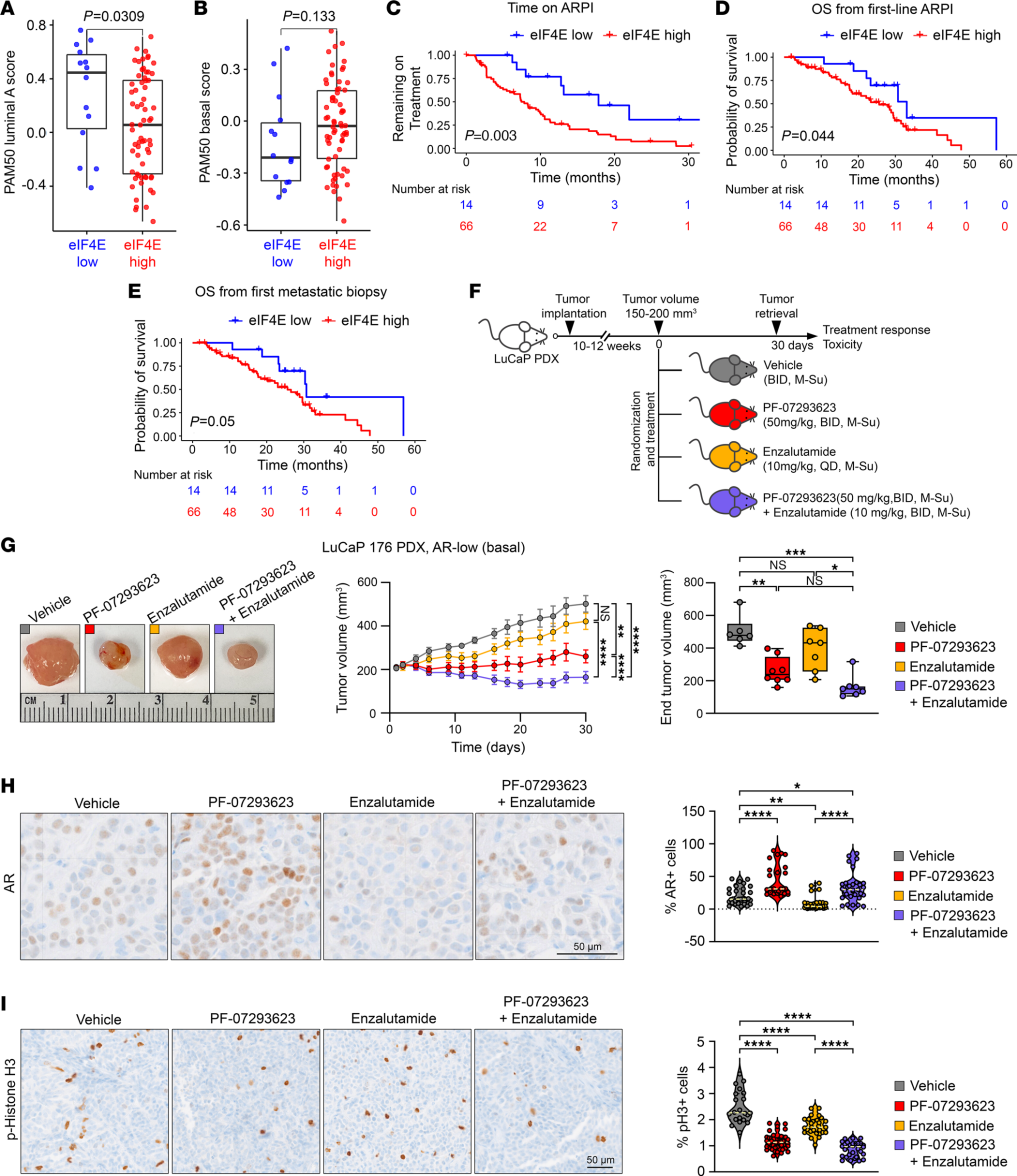
Cap binding domain of eIF4E drives lineage plasticity and restores sensitivity to ARPIs. (**A**–**E**) SU2C mCRPC patients stratified by eIF4E expression, *n* = 80: (**A**) Luminal A PAM50 scores, (**B**) Basal PAM50 scores, (**C**) Progression-free survival on ARPI, (**D**) OS from first-line ARPI, (**E**) OS from metastatic biopsy. (**F**) Preclinical trial schematic. (**G**) Representative LuCaP 176 PDX tumors (left), tumor growth curves (middle), and endpoint tumor volumes (right). *n* = 6 or more mice per group. (**H** and **I**) Representative IHC staining and quantification of 176 PDX tumor for (**H**) AR and (**I**) phospho-histone H3. Plots represent mean ± SEM. Significance was determined by Wilcoxon rank-sum tests in **A** and **B**; log-rank tests in **C**–**E**; by Brown-Forsythe and Welch ANOVA with Dunnett’s T3 multiple comparison tests in **G**; by 1-way ANOVA with Šidák’s multiple comparison tests in **H** and **I**. *P < 0.05; **P < 0.01; ***P < 0.001; ****P < 0.0001.

Given our mechanistic evidence that eIF4E drives lineage plasticity in CRPC, we next sought to determine if eIF4E mRNA levels associate with lineage features in patients. To this end, we analyzed data from the SU2C East Coast Dream Team (ECDT) composed of metastatic biopsies from patients with CRPC focusing on individuals who previously received an ARPI (*n* = 80) (53). Patients with tumors were stratified by low (below 25th percentile, *n* = 14) or high (all remaining patients, *n* = 66) expression of eIF4E in the SU2C cohort (Supplemental Figure 7A). We found that tumors with high eIF4E expression exhibited lower luminal scores and a trend towards higher basal scores, indicating that elevated eIF4E is associated with a basal-like, less luminal phenotype (Figure 6, A and B) (54). Importantly, patients with high eIF4E levels developed resistance to ARPIs including abiraterone, enzalutamide, or apalutamide, more rapidly than those with low eIF4E expression, supporting a potential role for eIF4E in driving therapy resistance in CRPC (Figure 6C). In addition, high eIF4E expression was associated with reduced overall survival (OS) (Figure 6, D and E). Univariate analysis of clinical features revealed a significant association between eIF4E expression and shorter time on ARPI, as well as reduced OS from initiation of first line ARPI (Supplemental Figure 7, B–D). Prior chemotherapy was also associated with reduced OS from first biopsy. In multivariate analysis of clinical features with *P* < 0.1 in the univariate analysis, both eIF4E expression and prior chemotherapy remained significantly associated with shorter time on ARPI and reduced OS from first line ARPI and from first biopsy (Supplemental Figure 7, B–D). These findings collectively suggest that high eIF4E expression is associated with a more basal-like molecular phenotype and represents a significant variable corresponding to a more aggressive and therapy-resistant disease state. Additionally, these findings highlight the therapeutic potential of targeting the eIF4E cap-binding domain in advanced prostate cancer with a basal phenotype to promote lineage plasticity toward a more luminal state, potentially reversing resistance and enhancing responsiveness to AR-targeted therapies.

### eIF4E cap-binding domain inhibition drives lineage plasticity and sensitization to enzalutamide in vivo.

Our patient-based and mechanistic data demonstrate that eIF4E correlates with and drives basal-to-luminal lineage plasticity. This raises the important question, can inhibition of the eIF4E cap-binding domain induce a lineage switch to sensitize AR-low, basal prostate cancers to ARPIs. To address this, we employed the LuCaP 176 PDX model (Figure 1L). Mice were randomized to receive vehicle, PF-07293623 (50 mg/kg, orally, twice daily), the ARPI enzalutamide (10 mg/kg, orally, daily), or a combination of PF-07293623 and enzalutamide (50 mg/kg and 10 mg/kg, respectively) (Figure 6F). As expected, the LuCaP 176 PDX was insensitive to enzalutamide (Figure 6G). However, combining PF-07293623 with enzalutamide led to tumor regression, which was not observed with either therapy alone (Figure 6G). Mice treated with PF-07293623 alone or in combination with enzalutamide showed no signs of toxicity (Supplemental Figure 7, E and F). In contrast, a subset of mice in the enzalutamide-only treatment group exhibited leukopenia, anemia, and thrombocytopenia, toxicities that have been reported in patients receiving enzalutamide (Supplemental Figure 7, E and F) (55). These results demonstrate that eIF4E cap-binding domain inhibition can sensitize resistant AR-low prostate cancer to enzalutamide.

To determine if PF-07293623 caused drug-induced lineage plasticity by converting AR-low LuCaP 176 tumors to a more luminal state, we assessed AR expression. Treatment with PF alone led to a significant increase in AR-positive cancer cells (Figure 6H). Moreover, the combination of enzalutamide and PF-07293623 resulted in higher AR protein abundance compared with enzalutamide alone, suggesting enhanced luminal features following eIF4E cap-binding domain inhibition (Figure 6H). Consistent with the 2D LuCaP 176 model, PF-0729362– treated LuCaP 176 tumors exhibited increased BAP1 and OTUD3 expression, deubiquitinases that may contribute to AR upregulation through protein stabilization (Figure 5G, Supplemental Figure 7G). Next, we sought to determine the cellular mechanisms underlying the reduction in tumor growth observed with PF-07293623 treatment. Phospho-histone H3 (pHH3) staining revealed decreased proliferative activity in tumors treated with enzalutamide, PF-07293623, and the combination. However, the most profound decrease in proliferation was observed in tumors treated with PF-07293623 and enzalutamide (Figure 6I). Additionally, PF07293623-treated tumors exhibited increased cellular apoptosis, supporting its role in promoting programmed cell death (Supplemental Figure 7H). Of note, no difference in necrosis was observed (Supplemental Figure 7I). Together, these data demonstrate that eIF4E cap-binding domain inhibition promotes lineage plasticity in AR-low basal prostate cancer in vivo, shifting it toward a luminal phenotype characterized by increased AR protein levels and enhanced sensitivity to an AR-targeted therapy.

## Discussion

Our study reveals that eIF4E maintains the basal lineage in prostate cancer. Though it has been shown that targeting eIF4E phosphorylation, or its interaction with cofactors such as eIF4G, can influence the transformative capacity of prostate epithelial cells or prostate cancer progression (24, 41), no studies to date have linked eIF4E directly to a lineage state. We believe that our study is unique because it unveils the critical role of the cap-binding domain of eIF4E in regulated mRNA specific translation of lineage markers. We discovered that a regulon of basal keratins possesses a unique cis-regulatory element within their 5′ UTRs (AGCCWCCAGC) that endows them with sensitivity to eIF4E cap-binding domain inhibition (Figure 4D). Without this motif, basal keratins were rendered insensitive to perturbations of eIF4E. Interestingly, this motif is fundamentally different from other eIF4E-sensitive 5′ UTR sequences, including the pyrimidine-rich translational element (PRTE) and the guanine-rich translational element (GRTE) (24, 56). It is interesting to speculate why different cis-regulatory elements exist downstream of eIF4E. One explanation is that each motif is dependent on different aspects of eIF4E function. For example, 5′ UTRs possessing PRTEs are sensitive to changes in eIF4E-4EBP1 interactions, while GRTE-containing 5′ UTRs are dependent on eIF4E-eIF4G interactions (24, 57). An additional explanation is that eIF4E regulates distinct translational programs at different steps of tumorigenesis. For example, eIF4E-mediated selective translation has been implicated in cancer initiation and metastatic progression (56), while eIF4E phosphorylation–mediated translational control may be especially important during early metabolic adaptation of tumor cells to enable proliferation and survival (58). In this study, we identify a distinct role of eIF4E 5′ cap-binding domain–mediated translational control in regulating lineage plasticity in cancer. It remains to be determined if additional cofactors are necessary to enable basal keratin-specific mRNA translation. Beyond the translation regulation of basal keratins by eIF4E, our work also demonstrates that these keratins are essential for the survival of prostate cancer. Thus, basal keratin are not only markers to differentiate lineage states, but are also necessary structural components of the cell critical for survival.

In addition to downregulation of basal keratins, we also observed a dramatic increase in AR protein levels upon inhibition of the eIF4E cap-binding domain, suggesting a lineage switch to a luminal phenotype. This is in contrast with observations demonstrating that eIF4A inhibition with eFT226 decreases AR levels (59). RNA-seq and HPG-TMT mass spectrometry analysis of AR suggested that the increase in AR was not mediated by a transcriptional or translational mechanism (Supplemental Figure 6, B and C). However, we found that proteins that were upregulated by eIF4E inhibition were enriched for posttranslational modifiers, suggesting a potential mechanism that could titrate AR levels (Supplemental Figure 6E). Multiple posttranslational modifications of AR, including phosphorylation by CDK1, acetylation by p300, methylation by EZH2, ubiquitination by SPOP, and SUMOylation by PIAS1, have been reported to influence AR’s stability, nuclear localization, and transcriptional activity (60). In our study, we found 2 deubiquitinases, BAP1 and OTUD3, that were translationally upregulated upon inhibition of the eIF4E cap-binding domain. BAP1 and OTUD3 have been characterized as tumor suppressors in prostate cancer due to their role in deubiquitinating and stabilizing PTEN (46, 48). In addition, previous studies have reported a positive correlation between BAP1 expression and AR levels in patients with prostate cancer (61, 62). However, their role in promoting AR stability remains unexplored. Through knockdown studies, we found that BAP1 and OTUD3 were essential for maintaining AR protein levels by limiting proteosome-mediated turnover (Figure 5, H–K). These findings raise the question of what might increase BAP1 and OTUD3 protein levels in the context of eIF4E inhibition. Our HPG-TMT mass spectrometry and RNA-seq data indicate that these proteins were synthesized more rapidly in the context of PF-07293623 treatment without any changes at the mRNA level. Two potential mechanisms that could explain their improved translation are the presence of upstream open reading frames (uORFs) or internal ribosome entry sights (IRESs) within their 5′ UTRs, which may enable selective translation of specific transcripts when canonical cap-dependent initiation is constrained; however, the contribution of these mechanisms remains to be determined (63, 64).

From a clinical-translational perspective, the nature of eIF4E inhibition is a critical question. Given the variety of mRNA species that can be differentially affected by altering eIF4E function, determining which ones are key weaknesses in clinically relevant models and patients will be important for advancing the field. In prostate cancer, there has been marked interest in targeting eIF4E phosphorylation by inhibiting the MNK kinases (65). A clinical grade MNK1/2 inhibitor (eFT508) was tested in individuals with CRPC (66) but was terminated early due to a lack of efficacy (https://clinicaltrials.gov/study/NCT03690141), despite being well tolerated. One interpretation of this clinical trial is that eIF4E is not a good therapeutic target in prostate cancer. However, our findings would suggest a need for deeper consideration. We found that eFT508 was ineffective at inhibiting CRPC growth (Figure 1C). Furthermore, even complete removal of eIF4E phosphorylation was insufficient to decrease cap binding within prostate epithelial cells (Figure 2G). These results indicate that, while targeting eIF4E phosphorylation may be ineffective, other domains of eIF4E, such as its cap-binding domain, may be critical for therapeutic intervention in prostate cancer. Supporting this, overexpression of eIF4E was able to rescue growth inhibition induced by cap-binding domain inhibition in prostate cancer cells, highlighting the functional importance of this domain and its therapeutic potential of targeting translation initiation (Figure 1I).

Lastly, our findings demonstrate the preclinical significance of a basal-to-luminal lineage conversion driven by eIF4E cap-binding domain inhibition. We found that ARPI efficacy can be improved with the administration of PF-07293623, rendering previously resistant tumors sensitive to enzalutamide (Figure 6G). Interestingly, we found that tumors of patients with CRPC that exhibit basal features were also more likely to express eIF4E at high levels and have shorter time to progression on ARPIs. This is consistent with observations in another cohort of patients with mCRPC, where individuals with luminal tumors treated with ARPIs had significantly better survival compared with patients with basal tumors (35). Our findings position cap-binding domain inhibitors as a therapy with the greatest potential in patients who have tumors that harbor basal phenotypes and are resistant to ARPIs. Notably, even among aggressive and therapy-resistant NEPC models, basal phenotype cells such as LTL331Rs, which express KRT5, were highly sensitive to eIF4E cap-binding domain inhibition, while nonbasal cells (MSKCC-EF1 and NCI-H660), which lack KRT5, remained largely unaffected (Supplemental Figure 8A) (67–69). To explore whether this concept extends beyond prostate cancer, we tested breast cancer cell lines of basal versus luminal phenotypes and observed a similar pattern where basal breast cancer cells were more sensitive to eIF4E cap-binding domain inhibition (Supplemental Figure 8B) (70). Collectively, these results support the idea that tumors with basal phenotypes, across cancer types, may be particularly vulnerable to targeting the eIF4E cap-binding domain. Understanding which specific basal features confer sensitivity to eIF4E cap-binding domain inhibition will be critical for uncovering the underlying mechanisms of this vulnerability across cancers. Such insights could also inform patient stratification, enabling the identification of individuals most likely to benefit from cap-binding domain targeted therapies.

## Methods

### Sex as a biological variable.

The murine system used in our study was exclusively male because prostate cancer is only relevant in males.

### Cell culture.

The cell lines used in this study included LuCaP 35CS (71), LuCaP 35CR (71), LuCaP 176 (72), PC3, DU145, LNCaP, 22Rv1, VCaP, Primary Prostate Epithelial Cells (PrEC), LTL331R (67), MSKCC-EF1 (68), NCI-H660, BT-549, HCC38 and MCF7. All cell lines were obtained from the American Type Culture Collection (ATCC), unless otherwise indicated in Supplemental Table 4. The LuCaP cell lines were authenticated by STR or genomic sequencing. All cell lines were maintained in the growth media (Supplemental Table 4) at 37°C in a humidified incubator with 5% CO_2_.

### Pharmacological screen.

Cells were seeded in 96-well plates at optimized densities in 200 μL of growth medium to maintain exponential growth. Each condition included three or more biological replicates with two or more technical replicates. Plates were incubated for 24 hours at 37 °C with 5% CO_2_ before treatment with compounds in a 10-point serial dilution (10 μM to 0.0001 μM) for 96 hours, with 0.3% (v/v) DMSO per well. Imaging was performed every 6 hours using the Cytation 5 (BioTek). Cell count and object sum area were quantified by image analysis and percentage growth was calculated as: (object sum area / total imaged area) × 100, normalized to vehicle controls. All drugs were obtained directly from companies except 2BAct (Aobious, cat. #AOB17667) and homoharringtonine (Abcam, cat. #ab142580).

### NucView apoptosis assay.

LuCaP 176 cells were seeded in 96-well plates (10,000 cells/well) and treated with PF-07293623 or DMSO. Apoptosis was induced with staurosporine (1 μM) and co-treatment with the caspase-3/7 inhibitor Ac-DEVD-CHO (10 μM) served as negative control. All wells received Caspase-3 substrate (NucView® 488, 5 μM; Biotium, cat. #30029) for visualization. Two independent experiments were performed, each with technical duplicates. Cells were imaged every 2 hours on Incucyte platform for 72 hours and green fluorescence intensity was analyzed as a measure of caspase-3 activation.

### eIF4E overexpression.

WT eIF4E was PCR-amplified from pDONR223_eIF4E_WT (Addgene, cat. #82112) and cloned into pLenti-CMV-GFP (Addgene, cat. #17448, also served as control) to generate pLenti-CMV-eIF4E-P2A-GFP. Lentivirus for these plasmids were produced in 293T cells using the CaCl_2_ transfection method. LuCaP 176 cells were Transduced and selected with puromycin (0.5 μg/mL) for 5 days to enrich GFP^+^ populations, which were seeded for growth assays with PF-07293623 or DMSO treatments. Experiments were performed three times with at least two technical replicates. Cells were imaged every 6 hours for 72 hours using Incucyte.

### Western blot.

Western blot analysis was performed as previously described (73) with antibodies listed in Supplemental Table 5. Blots were analyzed for band intensity and protein levels were normalized to vehicle or siNT using ImageJ.

### Cell cycle analysis.

Cells were fixed using pre-cooled 66% ethanol at 4°C for 2 hours. Propidium iodide staining was performed following manufacturer’s instruction (Abcam, cat. #ab139418). The cells were analyzed using a Fortessa X50 flow cytometer and cell cycle phases were analyzed using FlowJo v10.10.0.

### In vitro HPG labelling and assessment of protein synthesis rates.

Cells were incubated in methionine-free DMEM (Thermo Fisher, cat. #21013024) with 10% FBS for 40 minutes at 37°C, then labeled with 100 μM L-homopropargylglycine (HPG; Click Chemistry Tools, cat. #1067) for 30 minutes. Cell pellets were fixed in 4% paraformaldehyde (PFA) and permeabilized with 0.5% Triton X-100 for 10 minutes each. Click labeling was performed using the Click-iT Cell Reaction Buffer Kit (Invitrogen, cat. #C10269) with 5 μM Alexa Fluor 555-azide (Thermo Fisher, cat. #A20012) for 15 minutes at room temperature (RT). Labeled cells were washed twice and analyzed by flow cytometry (BD Symphony 2). Mean fluorescence intensity was quantified using FlowJo v10.10.0, normalized to controls and expressed as percentage HPG incorporation.

### Cap-binding assay.

Cap-binding assay was performed as previously described (24). m^7^GTP beads bound eIF4E was analyzed by western blot where flow-through fractions served as loading controls.

### HPG-TMT mass spectrometry.

Cells were treated with PF-07293623 (100 nM, 6 h) or DMSO in triplicate with HPG labeling during the final hour. Protein was extracted and measured by Bradford assay. 200 μg protein was conjugated with 40 μM biotin-azide (Thermo Fisher, cat. #10184) using Click-iT Protein Reaction Buffer Kit (Thermo Fisher, cat. #10276), precipitated and desalted (Zeba spin columns, Thermo Fisher, cat. #89882). 100 μg protein was incubated with streptavidin beads (Pierce, cat. #88816) overnight at 4 °C. Samples were processed at the Fred Hutchinson Cancer Center Proteomics Core where samples were reduced, alkylated, digested with Lys-C and trypsin, desalted, and labeled using TMT6plex. Peptides were pooled, fractionated into 24 fractions by basic reverse-phase chromatography, and analyzed by LC-MS/MS on an Orbitrap Eclipse (Thermo Scientific). Mass spectrometry data were analyzed using Proteome Discoverer v3.1, with peptide FDR < 1%. Raw reporter ion abundances were log_2_-transformed and median-normalized across samples. *P* values for pairwise comparisons were calculated by *t* test. Candidates were selected using a log_2_ fold change threshold of ± 0.13 (equivalent to a fold change of 1.1) and a *P* value < 0.05. MsigDB and Enrichr was used for pathway enrichment analysis. Heatmaps and dotplots were generated with pheatmap and volcano plots with ggplot2 in R. Mass spectrometry data analysis is provided in Supplemental Table 1.

### RNA-seq.

AR-low LuCaP 176 cells were treated with PF-07293623 (100 nM, 6 hours) or DMSO in triplicate. RNA was extracted using the RNeasy Plus MinElute kit (Qiagen, cat. #74134) and assessed for concentration and quality on the Agilent 4200 TapeStation. ERCC RNA Spike-In Control Mix (Thermo Fisher, cat. #4456740) was diluted (1:1000), and 2 μL was added per 100 ng of RNA. Libraries were prepared with TruSeq Stranded mRNA Library Prep Kit (Illumina, cat. #20020595) and indexed with TruSeq RNA CD Index Plate (Illumina, cat. #20019792) as per manufacturer’s protocol. Sequencing was performed on an Illumina NextSeq platform with paired-end reads at the Fred Hutchinson Cancer Center Genomics Core. Raw reads were quality checked using FastQC (https://www.bioinformatics.babraham.ac.uk/projects/fastqc/), aligned to UCSC mm10 and quantified using HTSeq. Normalized counts were used for principal component analysis (PCA) in R and differential expression was analyzed with edgeR using a log_2_ fold change threshold of ±0.13 and a false discovery rate (FDR) < 0.05 to identify transcriptionally regulated genes. RNA-seq analysis is shown in Supplemental Table 2.

### siRNA knockdown.

siRNA knockdowns was performed in AR-Low LuCaP 176 cells as previously described (73) using SMARTpool of siRNAs (Dharmacon) targeting BAP1 (cat. #L-005791-00-0005), KRT2 (cat. #L-011066-00-0005), KRT5 (cat. #L-011067-00-0005), KRT6B (cat. #L-012117-02-0005), and KRT9 (cat. #L-011068-00-0005), OTUD3 (cat. #L-027582-00-0005) or pooled knockdown (siKRT pool) with siRNAs targeting *KRT2*, *KRT5*, *KRT6B* and *KRT9* or a non-targeting control pool of siRNA (cat. #D-001810-10-05).

### Cell-free and in-cell SHAPE-MaP of KRT5 5′ UTR.

LuCaP 176 cells were treated with 5-nitroisatoic anhydride (5NIA) (CAS No. 4693-02-1; AstaTech Inc, cat. #69445), and SHAPE experiments were performed and analyzed as previously described (43). The *KRT5* 5′ UTR (NM_000424.4, 98 bp) was amplified from RNA using primers (Forward: 5′-AACAGAGCCACCTTCTGCGT-3′, Reverse: 5′-GAAGCTACGACTGCCCCCG-3′). Illumina adapters (Illumina, cat. #200015964/5) were incorporated during library preparation. Libraries were purified using Mag-Bind Total Pure NGS beads (Omega Bio-Tek, cat. #75877-716) and assessed on an Agilent TapeStation. Libraries were pooled and sequenced on an Illumina MiSeq at the Fred Hutchinson Genomics Core. FASTQ files were processed with ShapeMapper2 using a minimum read depth of 5000. Replicates were averaged in R, and profiles were aligned to the *KRT5* mRNA transcript. Secondary structure models and base-pairing probabilities were generated using Superfold and RNAvigate.

### 5′ UTRs motif discovery.

The 5′ untranslated regions (5′ UTRs) of *KRT2*, *KRT 5*, *KRT6B*, *KRT9*, *KRT14* and *KRT71* were analyzed for conserved sequence motifs using the STREME in the MEME Suite. 5′ UTRs of transcripts corresponding to HPG-TMT targets were scanned for motif occurrences using FIMO in the MEME Suite.

### KRT5 5’ UTR luciferase assay.

The KRT5 WT, KRT5 SL-DEL and KRT5 DEL 5′ UTR luciferase reporter constructs were cloned by inserting either the full-length (1-98 position) or 41-69 deletion (CCAGCACCTCCCAACCCACTAGTGCCTGG) or 6-15 cis-element deletion (AGCCACCUUC) into a CMV-Luc2CP backbone (Addgene, plasmid #62857) immediately upstream of the firefly luciferase of pLuc2CP-noARE plasmid using Gibson Assembly Master Mix (NEB, cat. #E2611S). Dual-luciferase assay was performed on AR-low LuCaP 176 cells as previously described (73). Firefly luciferase activity was measured on Cytation 5 (BioTek) and normalized to luciferase mRNA to quantify translational changes.

### qRT-PCR.

qRT-PCR was performed as previously described (24). The primers used are listed in Supplemental Table 6.

### AR CUT&RUN.

CUT&RUN was performed and analyzed in triplicate using 4 × 10^6^ LuCaP 176 cells per reaction from PF-07293623 or DMSO treated cells cultured for 48 hours in 10% charcoal-stripped serum (Gibco, cat. #12676029), as previously described (74). Briefly, ConA paramagnetic beads (EpiCypher, cat. #21-1401) were activated, and cells were immobilized on the beads, washed, and incubated overnight at 4 °C with anti-AR antibody or normal rabbit IgG (see Supplemental Table 5) diluted in wash buffer containing 0.1% Triton X-100. Following antibody binding, samples were processed at the Fred Hutchinson Cancer Center Genomics Shared Resource. Libraries were prepared using a Beckman Biomek i7 liquid handling instrument with a 96S Super Magnet Plate (Alpaqua SKU A001322). MNase digestion was performed at 4 °C and stopped after 120 minutes with EGTA, followed by end repair, adapter ligation, PCR amplification, and size assessment on an Agilent TapeStation. Libraries were pooled at equimolar concentrations and sequenced (paired-end 50 x 50 bp) on an Illumina NovaSeq X Plus. Reads were aligned to the hg38 genome using Bowtie 2. Peaks were called using SEACR. Gene annotations were performed using HOMER and the ChIPSeeker R package. Differentially bound peaks were determined using the DiffBind R package. Peaks were visualized using IGV. DeepTools 3.3.0 was used to calculate matrices and plot heatmaps for binding sites.

### AR ChIP.

ChIP assays were performed in triplicate using 4 × 106 LuCaP 176 cells per reaction (PF-07293623 or DMSO control) with the Magnetic ChIP Kit (Pierce, cat. #26157). Immunoprecipitations were carried out using anti-AR antibody (see Supplemental Table 5) or normal rabbit IgG (Pierce Magnetic ChIP Kit, cat. #26157) as control. ChIP-qPCR was performed using immunoprecipitated DNA (9.45 ng) and input DNA (18.90 ng) with SsoAdvanced Universal SYBR Green Supermix (Bio-Rad, cat. #1725271). Data were analyzed using the ΔCq (ΔCq = Cq [specific antibody] − Cq [normal IgG]) and reported as fold enrichment (2^–^ΔCq) over IgG. Primers are listed in Supplemental Table 6.

### Preclinical trial.

Tumor pieces (1 × 1 × 1 mm) were subcutaneously implanted into the flanks of 10-12 week old male NSG mice (strain #005557, RRID: IMSR_JAX:005557). Tumor growth and mouse weights were monitored three times weekly. Tumor volume was calculated as: l/2 × w^2^. When tumors reached 150-200 mm³ volume, mice (6 or more per group) were randomized to receive: PF-07293623 (50 mg/kg, BID), enzalutamide (10 mg/kg, QD), combination therapy, or vehicle controls, administered via oral gavage for 30 days. Single-agent groups received the corresponding alternate vehicle. Formulations were freshly prepared weekly. No attrition occurred, and treatments were not blinded.

### Cell titer glo.

Cell viability was assessed using the CellTiter Glo 2.0 Assay (Promega, cat. #G9242) per manufacturer instructions. Cells were seeded in T-75 flasks and treated with PF-07293623 (100 nM, 72 hour) or DMSO. Cells were pelleted, mixed with 100 μL of CellTiter-Glo reagent (1:1) in a 96-well plate, incubated for 10 minutes at RT, and luminescence was measured using Cytation 5 (BioTek). Assays were performed in triplicate, and viability was calculated as fold change relative to DMSO control.

### Patient data analysis.

Bulk flash-frozen needle biopsies from the SU2C ECDT cohort, LuCaP PDX tumors, and cell lines were sequenced and aligned as described previously (54). Gene level abundance was quantified with GenomicAlignments and log_2_ FPKM were calculated. Single-sample enrichment scores were computed using GSVA with genome-wide log_2_ FPKM. Pearson correlations were calculated using cor.test. Kaplan-Meier curves were estimated with survfit and plotted using survminer. AR+/NE+ and AR-/NE+ tumors were excluded. Tumors were stratified by high (>25th percentile) or low (<25th percentile) eIF4E expression. Univariate and multivariate Cox analyses were performed with coxph, with multivariate models including only variables with univariate *P* ≤ 0.1, and results plotted using forest_model. Differences between survival curves were tested with the log-rank test. The LuCaP PDX RNAseq data used in this study are available in the Gene Expression Omnibus repository (GEO) under accession number GSE199596. SU2C-IDT/PCF RNAseq data are available in the cBioPortal (prad_su2c_2019; https://github.com/cBioPortal/datahub/tree/master/public/prad_su2c_2019.)

### Statistics.

All statistical analysis was done either in GraphPad Prism (version 9) or R (v4.2.0) using unpaired 2-tailed Student’s *t* test, 1-way ANOVA with multiple comparisons, or Wilcoxon’s rank-sum tests, unless otherwise specified. Sample sizes are provided in the Methods. *P* < 0.05 was considered statistically significant.

### Study approval.

All animal experiments were performed in compliance with animal care guidelines approved by the Institutional Animal Care and Use Committee (IACUC) at the Fred Hutchinson Cancer Center.

### Data Availability.

The RNA and CUT&RUN sequencing data files are publicly available on the GEO database at GSE301507 and GSE318825, respectively. Mass spectrometry and proteomics data files are available on the MassIVE repository at MSV000098490. The code for data processing and analysis is on GitHub (https://github.com/sonali-bioc/Mishra_eiF4E_lineage_plasticity). All other data are provided in the Supplemental tables. Values for all graph data points are reported in the Supporting Data Values XLS file.

## Author contributions

RM and ACH conceived and designed the study. RM, SS, DC, DR, CLW, PH, JYK, SD, AEO, SK, and AS performed experiments and data analysis. IMC, SA, CL and HL contributed to formal analysis and data curation. PRG, EC, and PSN provided supervision and resources. PSN and ACH provided funding support. RM and ACH wrote the manuscript, with review and editing contributions from all authors.

## Conflict of interest

EC served as a paid consultant to DotQuant and received Institutional sponsored research funding unrelated to this work from Astra Zeneca, AbbVie, Gilead, Sanofi, Zenith Epigenetics, Bayer Pharmaceuticals, Forma Therapeutics, Genentech, GSK, Janssen Research, Kronos Bio, Foghorn Therapeutics, K36 Therapeutics, and MacroGenics. ACH serves on the scientific advisory board of Interdict Bio.

## Funding support

This work is the result of NIH funding, in whole or in part, and is subject to the NIH Public Access Policy. Through acceptance of this federal funding, the NIH has been given a right to make the work publicly available in PubMed Central. This work was supported by:

- NIH grants R37 CA230617, R01 GM135362, R01 CA276308 (ACH).
- Prostate Cancer Foundation Challenge Award (ACH).
- American Cancer Society Discovery Boost Award (DBG-25-1373505-01-RMC).
- Seattle Translational Tumor Research.
- The Nancy & Dick Bernheimer Memorial Fund (ACH).
- The Matthews Family Memorial Fund (ACH).
- The Stinchcomb Family Memorial Fund (ACH).
- The Thomas & Patricia Wright Memorial Fund (ACH).
- The Larry & Virginia Gordon Endowed Chair in Prostate and Bladder Cancer Research (ACH).
- RM was supported by the Fred Hutch Interdisciplinary Training Grant and NIH NCI P50 CA097186.
- Genomics, Proteomics, Cellular Imaging, Histopathology, Comparative Medicine and Bioinformatics Shared Resources of the FHCC (P30 CA015704).
- NIH P50 CA097186 (PSN).
- The Institute for Prostate Cancer Research (PSN).
- NIH grants R01CA266452, PC230420, P01CA298991, and P01CA163227 (PSN).
- IMC is also supported by these grants and by NIH NCI grant R50 CA274336 (IMC).
- Prostate Cancer Foundation Young Investigator Award (HL).
- Pilot grant from the Mike Slive Foundation for Prostate Cancer Research (HL).
- National Science Foundation 2310684 (AS).

## Supplementary Material

Supplemental data

Unedited blot and gel images

Supplemental table 1

Supplemental table 2

Supplemental table 3

Supplemental table 4

Supplemental table 5

Supplemental table 6

Supporting data values

## Version 1 04/15/2026

In-Press Preview

## Version 2 06/15/2026

Electronic publication
