# Supplementary material for: Therapeutic targeting of the eIF4E cap-binding domain reveals control of lineage fate in prostate cancer
Source: J Clin Invest. 2026 Apr 14;136(12):e199838. doi: 10.1172/JCI199838 (PMC13262746; doi:10.1172/JCI199838)
Supplement: Supplemental data [file jci-136-199838-s107.pdf]

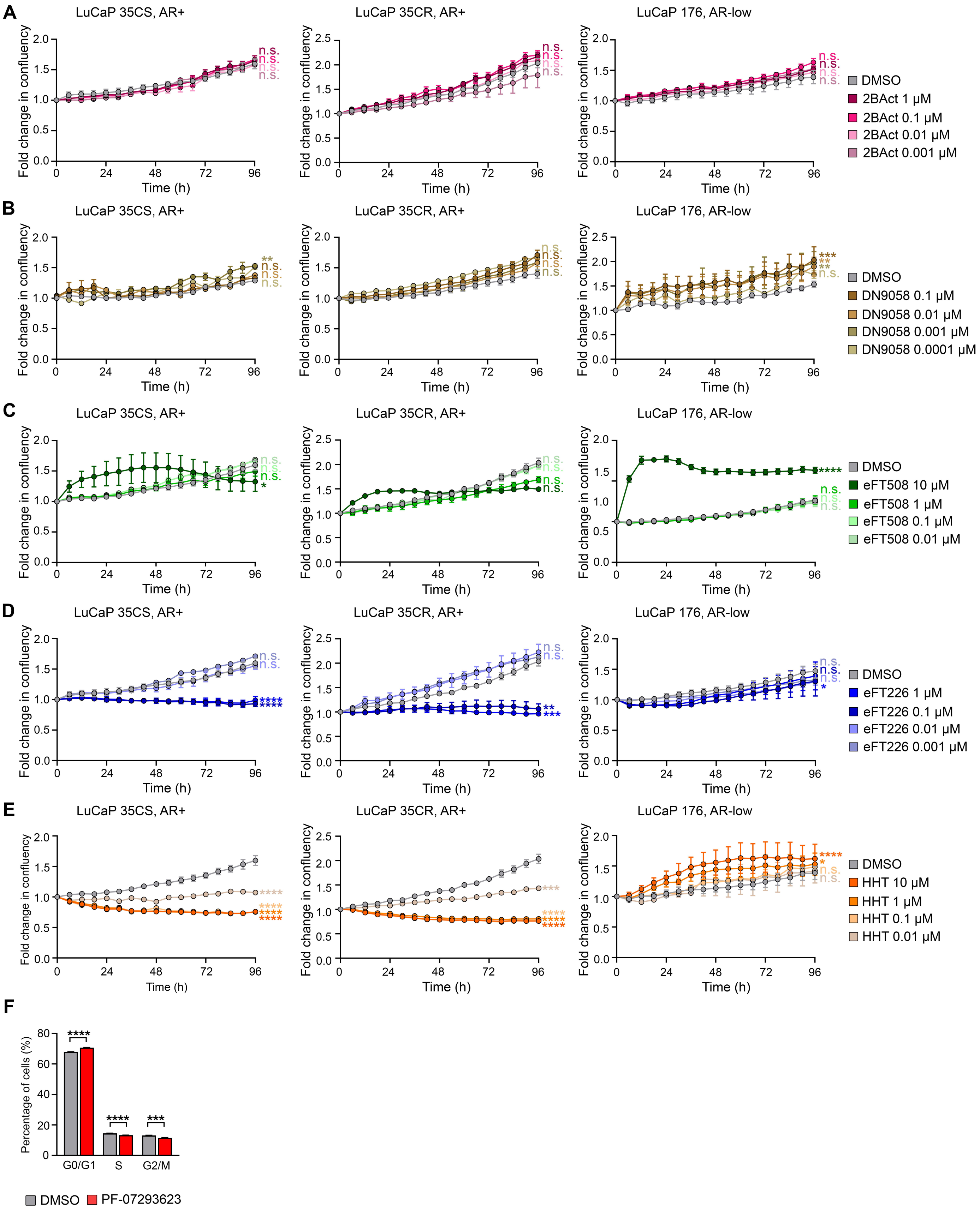
A
LuCaP 35CS, AR+
LuCaP 35CR, AR+
LuCaP 176, AR-low
Fold change in confluency
Time (h)
n.s.
DMSO
2BAct 1 μM
2BAct 0.1 μM
2BAct 0.01 μM
2BAct 0.001 μM
B
LuCaP 35CS, AR+
LuCaP 35CR, AR+
LuCaP 176, AR-low
Fold change in confluency
Time (h)
**
***
n.s.
DMSO
DN9058 0.1 μM
DN9058 0.01 μM
DN9058 0.001 μM
DN9058 0.0001 μM
C
LuCaP 35CS, AR+
LuCaP 35CR, AR+
LuCaP 176, AR-low
Fold change in confluency
Time (h)
n.s.
*
****
DMSO
eFT508 10 μM
eFT508 1 μM
eFT508 0.1 μM
eFT508 0.01 μM
D
LuCaP 35CS, AR+
LuCaP 35CR, AR+
LuCaP 176, AR-low
Fold change in confluency
Time (h)
n.s.
****
**
***
*
DMSO
eFT226 1 μM
eFT226 0.1 μM
eFT226 0.01 μM
eFT226 0.001 μM
E
LuCaP 35CS, AR+
LuCaP 35CR, AR+
LuCaP 176, AR-low
Fold change in confluency
Time (h)
****
***
*
n.s.
DMSO
HHT 10 μM
HHT 1 μM
HHT 0.1 μM
HHT 0.01 μM
F
Percentage of cells (%)
****
***
G0/G1
S
G2/M
DMSO
PF-07293623

Supplemental Figure Legends

Supplemental Figure 1. mRNA translation inhibition suppresses growth in prostate cancer.

(**A**) Cell growth curves of 2BAct treated LuCaP 35CS, LuCaP 35CR, and LuCaP 176 cells normalized to vehicle group. (**B**) Cell growth curves of DN9058 treated LuCaP 35CS, LuCaP 35CR, and LuCaP 176 cells normalized to vehicle group. (**C**) Cell growth curves of eFT508 treated LuCaP 35CS, LuCaP 35CR, and LuCaP 176 cells normalized to vehicle group. (**D**) Cell growth curves of eFT226 treated LuCaP 35CS, LuCaP 35CR, and LuCaP 176 cells normalized to vehicle group. and (**E**) Cell growth curves of HHT treated LuCaP 35CS, LuCaP 35CR, and LuCaP 176 cells normalized to vehicle group. (**F**) Cell cycle analysis by PI staining upon DMSO (vehicle) or PF-07293623 treatment (100 nM, 72 hours) in LuCaP 176 cells. All plots represent mean ± SEM. Statistical significance was determined by one-way ANOVA with Dunnett's multiple comparisons test in **A-E**, n=3, by Unpaired two-tailed Student's *t*-test in **F**, n=3. n.s. = not significant ($p > 0.05$); *$p < 0.05$; **$p < 0.01$; ***$p < 0.001$; ****$p < 0.0001$.

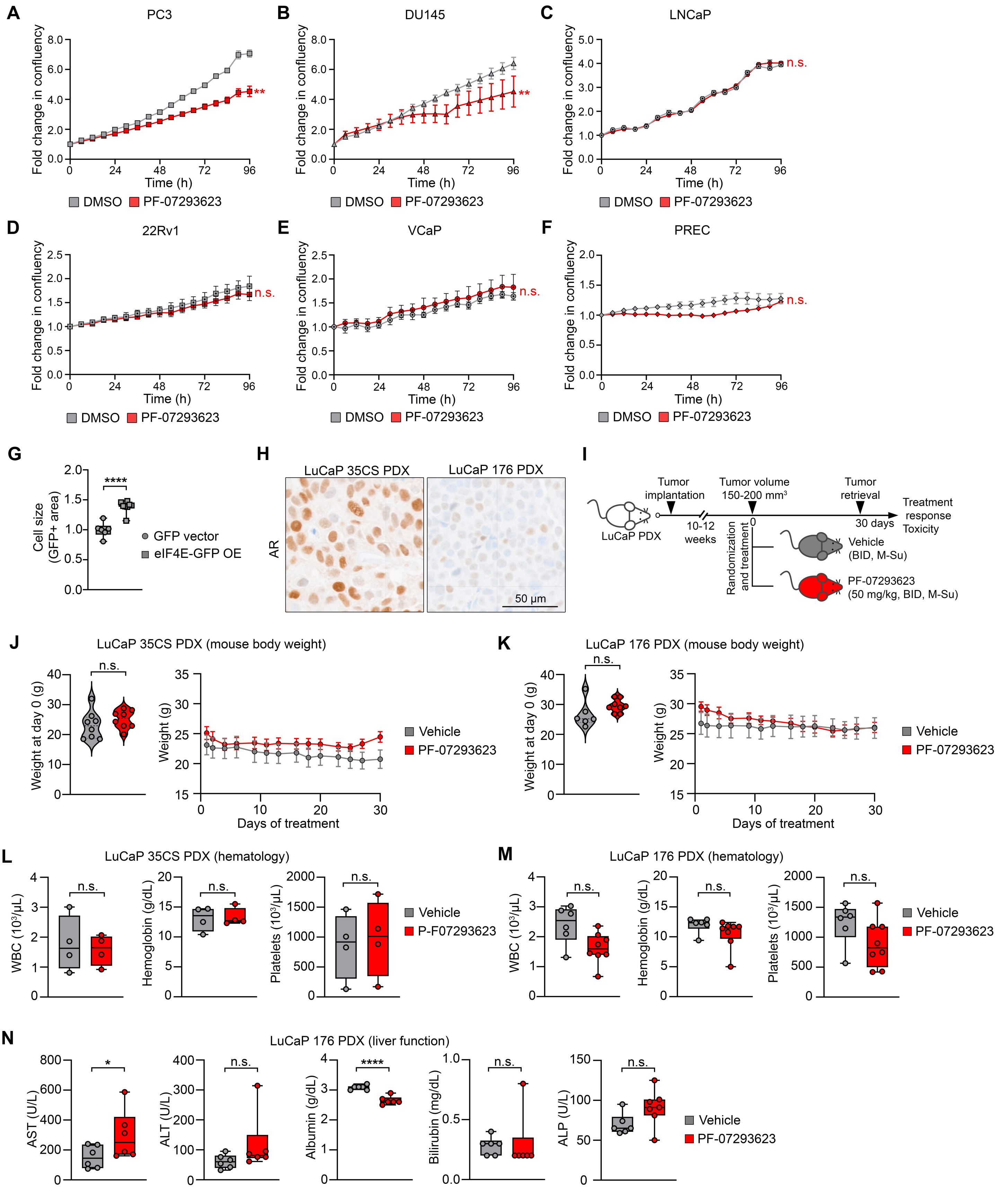
A
PC3
Fold change in confluency
Time (h)
DMSO
PF-07293623
**
B
DU145
Fold change in confluency
Time (h)
DMSO
PF-07293623
**
C
LNCaP
Fold change in confluency
Time (h)
DMSO
PF-07293623
n.s.
D
22Rv1
Fold change in confluency
Time (h)
DMSO
PF-07293623
n.s.
E
VCaP
Fold change in confluency
Time (h)
DMSO
PF-07293623
n.s.
F
PREC
Fold change in confluency
Time (h)
DMSO
PF-07293623
n.s.
G
Cell size (GFP+ area)
****
GFP vector
eIF4E-GFP OE
H
LuCaP 35CS PDX
LuCaP 176 PDX
AR
50 μm
I
LuCaP PDX
Tumor implantation
10-12 weeks
Tumor volume 150-200 mm³
0
Randomization and treatment
Tumor retrieval
30 days
Treatment response Toxicity
Vehicle (BID, M-Su)
PF-07293623 (50 mg/kg, BID, M-Su)
J
LuCaP 35CS PDX (mouse body weight)
Weight at day 0 (g)
n.s.
Weight (g)
Days of treatment
Vehicle
PF-07293623
K
LuCaP 176 PDX (mouse body weight)
Weight at day 0 (g)
n.s.
Weight (g)
Days of treatment
Vehicle
PF-07293623
L
LuCaP 35CS PDX (hematology)
WBC (10³/μL)
Hemoglobin (g/dL)
Platelets (10³/μL)
n.s.
Vehicle
P-F07293623
M
LuCaP 176 PDX (hematology)
WBC (10³/μL)
Hemoglobin (g/dL)
Platelets (10³/μL)
n.s.
Vehicle
PF-07293623
N
LuCaP 176 PDX (liver function)
AST (U/L)
ALT (U/L)
Albumin (g/dL)
Bilirubin (mg/dL)
ALP (U/L)
*
n.s.
****
Vehicle
PF-07293623

Supplemental Figure S2. mRNA translation inhibition suppresses growth in prostate cancer 2D and LuCaP PDX models.

(**A**) Cell growth curves of PF-07293623 (100 nM) treated PC3 cells normalized to vehicle. (**B**) Cell growth curves of PF-07293623 (100 nM) treated DU145 cells normalized to vehicle. (**C**) Cell growth curves of PF-07293623 (100 nM) treated LNCaP cells normalized to vehicle. (**D**) Cell growth curves of PF-07293623 (100 nM) treated 22Rv1 cells normalized to vehicle. (**E**) Cell growth curves of PF-07293623 (100 nM) treated VCaP cells normalized to vehicle. and (**F**) Cell growth curves of PF-07293623 (100 nM) treated PREC cells normalized to vehicle. (**G**) Cell size, quantified by GFP+ cell area in cells expressing GFP vector control or eIF4E-GFP overexpression constructs. (**H**) Immunohistochemical staining of AR on LuCaP 35CS and LuCaP 176 xenograft tumors. Scale bar = 50 µm. (**I**) Preclinical trial schematic. When tumor volumes reached 150–200 mm^3, mice were randomized and treated with vehicle (LuCaP 35CS n=6; LuCaP 176 n=6) or PF-07293623 (LuCaP 35CS n=7; LuCaP 176 n=8). (**J**) Mouse body weights of LuCaP 35CS PDX at start of the trial (left) and over the 30-day treatment period (right). (**K**) Mouse weights of LuCaP 176 PDX at start of the trial (left) and over the 30-day treatment period (right). (**L**) White blood cell, hemoglobin and platelet counts of LuCaP 35CS mice on trial. (**M**) White blood cell, hemoglobin and platelet counts of LuCaP 176 PDX mice on trial. (**N**) Serum AST, ALT, albumin, total bilirubin and ALP levels of LuCaP 176 PDX mice on trial. All plots represent mean ± SEM. Statistical significance was determined using a two-tailed t-test. n.s. = not significant ($p > 0.05$); *$p < 0.05$; **$p < 0.01$; ****$p < 0.0001$.

A

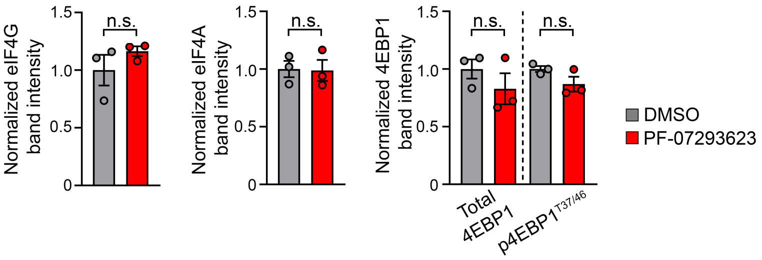
Normalized eIF4G band intensity
1.5
1.0
0.5
0
n.s.
Normalized eIF4A band intensity
n.s.
Normalized 4EBP1 band intensity
n.s.
n.s.
Total 4EBP1
p4EBP1T37/46
DMSO
PF-07293623

B

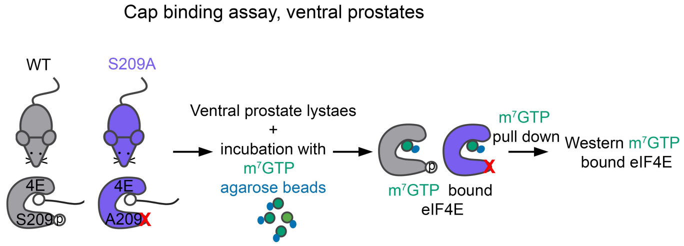
Cap binding assay, ventral prostates
WT
S209A
4E
S209
4E
A209
Ventral prostate lystaes
+
incubation with
m7GTP
agarose beads
m7GTP
bound
eIF4E
m7GTP
pull down
Western m7GTP
bound eIF4E

Supplemental Figure S3. eIF4E cap-binding domain inhibition suppresses translation by reducing its affinity to the m^{7}G cap.

(**A**) Quantification of band intensity of eIF4G, eIF4A, 4EBP1 and phospho-4EBP1$^{T37/46}$ in LuCaP 176 cells treated with DMSO or PF-07293623 (100 nM, 72 hours, n=3). All plots represent mean ± SEM. Statistical significance was determined using a two-tailed t-test. n.s. = not significant ($p > 0.05$). (**B**) Schematic of cap-binding assay on ventral lobes of prostates from WT or eIF4E S209A mutant mice.

A

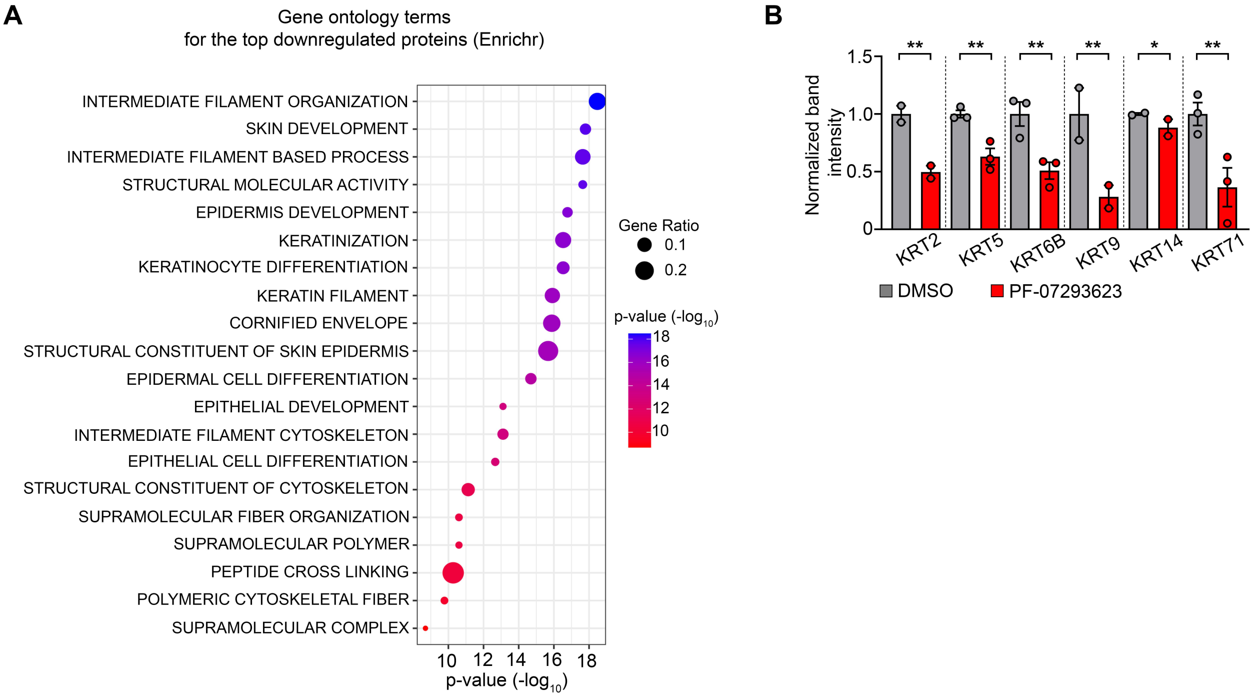
A
Gene ontology terms
for the top downregulated proteins (Enrichr)
INTERMEDIATE FILAMENT ORGANIZATION
SKIN DEVELOPMENT
INTERMEDIATE FILAMENT BASED PROCESS
STRUCTURAL MOLECULAR ACTIVITY
EPIDERMIS DEVELOPMENT
KERATINIZATION
KERATINOCYTE DIFFERENTIATION
KERATIN FILAMENT
CORNIFIED ENVELOPE
STRUCTURAL CONSTITUENT OF SKIN EPIDERMIS
EPIDERMAL CELL DIFFERENTIATION
EPITHELIAL DEVELOPMENT
INTERMEDIATE FILAMENT CYTOSKELETON
EPITHELIAL CELL DIFFERENTIATION
STRUCTURAL CONSTITUENT OF CYTOSKELETON
SUPRAMOLECULAR FIBER ORGANIZATION
SUPRAMOLECULAR POLYMER
PEPTIDE CROSS LINKING
POLYMERIC CYTOSKELETAL FIBER
SUPRAMOLECULAR COMPLEX
10 12 14 16 18
p-value (-log10)
Gene Ratio
0.1
0.2
p-value (-log10)
18
16
14
12
10
B
Normalized band intensity
1.5
1.0
0.5
0
** ** ** ** * **
KRT2
KRT5
KRT6B
KRT9
KRT14
KRT71
DMSO
PF-07293623

Supplemental Figure S4. Loss of basal keratins on eIF4E cap-binding domain inhibition.

(**A**) GSEA of gene ontology terms (GO, Enrichr) for the top 50 downregulated proteins upon PF-07293623 treatment. Dot size indicates- gene ratio; color reflects -$\log_{10}$ p-value. (**B**) Western blot quantification for KRT2, KRT5, KRT6B, KRT9, KRT14 and KRT71 in LuCaP 176 cells treated with PF-07293623 treatment (100 nM, 72 hours), normalized to vehicle group. All plots represent mean ± SEM. Statistical significance was determined using a two-tailed t-test. *$p < 0.05$; **$p < 0.01$.

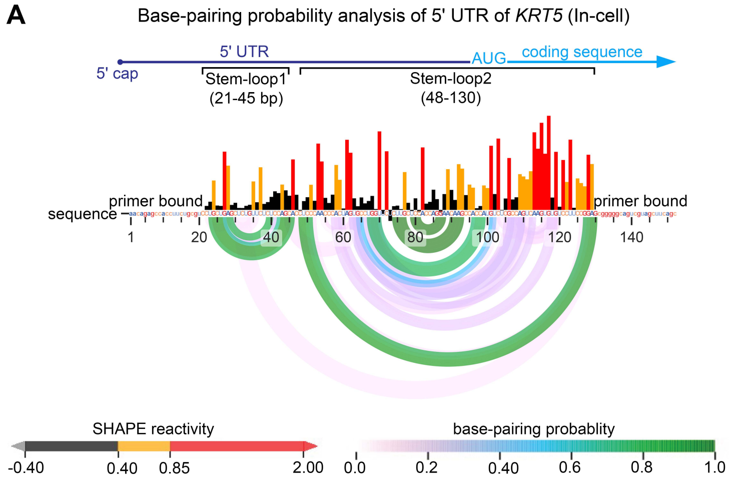
A
Base-pairing probability analysis of 5' UTR of KRT5 (In-cell)
5' UTR
5' cap
AUG
coding sequence
Stem-loop1
(21-45 bp)
Stem-loop2
(48-130)
primer bound
sequence
primer bound
1
20
40
60
80
100
120
140
SHAPE reactivity
-0.40
0.40
0.85
2.00
base-pairing probablity
0.0
0.2
0.40
0.6
0.8
1.0
B
Genes with 5′ UTR AGCCWCCAGC motif
Number of genes
Motif occurence
motif occurence
40.00
92.74
215.00
top HPG-TMT targets
upregulated
downreulated

Supplemental Figure S5. In-cell base-pairing probability of the *KRT5* 5' UTR and motif analysis of 5' UTR AGCCWCCAGC containing transcripts.

(**A**) Arc plot showing base-pairing probability analysis of the 5' UTR of *KRT5* using in-cell SHAPE reactivity. (**B**) Number of genes with AGCCWCCAGC motif and its frequency of occurrence within 5′ UTRs of upregulated and downregulated genes in LuCaP176 upon cap-binding domain inhibition, based on HPG-TMT dataset. Dot size indicates frequency of motif occurrence.

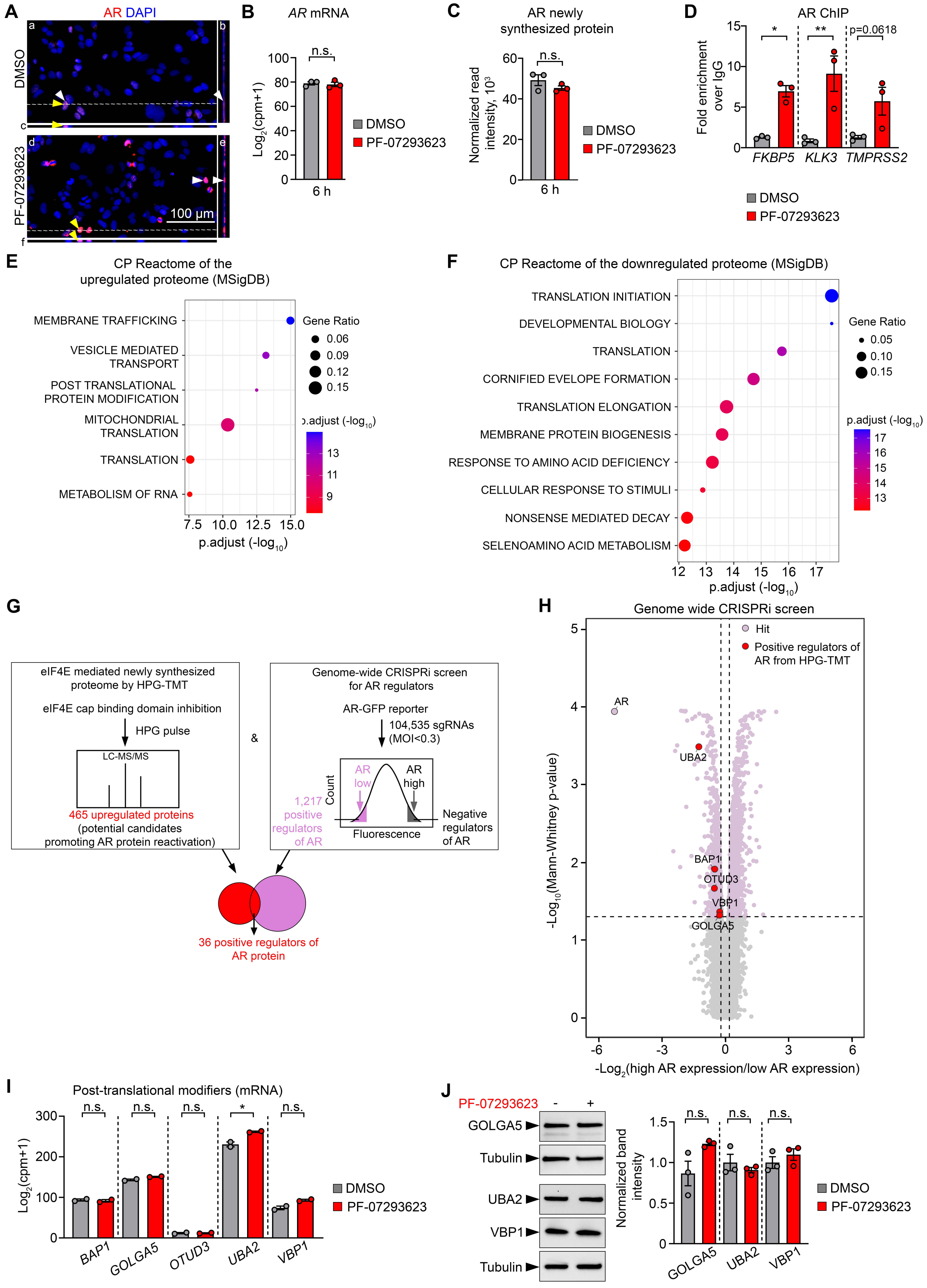
A
AR DAPI
DMSO
PF-07293623
100 μm
B
AR mRNA
n.s.
Log2(cpm+1)
6 h
DMSO
PF-07293623
C
AR newly synthesized protein
n.s.
Normalized read intensity, 10³
6 h
D
AR ChIP
Fold enrichment over IgG
p=0.0618
FKBP5
KLK3
TMPRSS2
E
CP Reactome of the upregulated proteome (MSigDB)
MEMBRANE TRAFFICKING
VESICLE MEDIATED TRANSPORT
POST TRANSLATIONAL PROTEIN MODIFICATION
MITOCHONDRIAL TRANSLATION
TRANSLATION
METABOLISM OF RNA
Gene Ratio
p.adjust (-log10)
F
CP Reactome of the downregulated proteome (MSigDB)
TRANSLATION INITIATION
DEVELOPMENTAL BIOLOGY
TRANSLATION
CORNIFIED EVELOPE FORMATION
TRANSLATION ELONGATION
MEMBRANE PROTEIN BIOGENESIS
RESPONSE TO AMINO ACID DEFICIENCY
CELLULAR RESPONSE TO STIMULI
NONSENSE MEDIATED DECAY
SELENOAMINO ACID METABOLISM
Gene Ratio
p.adjust (-log10)
G
eIF4E mediated newly synthesized proteome by HPG-TMT
eIF4E cap binding domain inhibition
HPG pulse
LC-MS/MS
465 upregulated proteins
(potential candidates promoting AR protein reactivation)
&
Genome-wide CRISPRi screen for AR regulators
AR-GFP reporter
104,535 sgRNAs (MOI<0.3)
AR low
AR high
Count
Fluorescence
1,217 positive regulators of AR
Negative regulators of AR
36 positive regulators of AR protein
H
Genome wide CRISPRi screen
Hit
Positive regulators of AR from HPG-TMT
AR
UBA2
BAP1
OTUD3
VBP1
GOLGA5
-Log10(Mann-Whitney p-value)
-Log2(high AR expression/low AR expression)
I
Post-translational modifiers (mRNA)
Log2(cpm+1)
BAP1
GOLGA5
OTUD3
UBA2
VBP1
J
PF-07293623
GOLGA5
Tubulin
UBA2
VBP1
Tubulin
Normalized band intensity
GOLGA5
UBA2
VBP1
DMSO
PF-07293623

Supplemental Figure S6. Identification of post-translational modifiers that stabilize AR upon eIF4E cap-binding domain inhibition.

(**A**) Fluorescent immunohistochemistry of AR (red) with DAPI (blue) in LuCaP 176 cells. Nuclear AR localization is shown in cells treated with DMSO or PF-07293623, visualized by z-stack analysis (white arrows, panels a & b; d & e) and 3D profiling (yellow arrows, panels a & c; d & f). (**B**) *AR* mRNA transcript levels in cells treated with DMSO or PF-07293623 (100 nM, 6hours, n=3). (**C**) Nascent AR protein expression intensity by HPG-TMT mass spectrometry of LuCaP 176 cells treated with DMSO or PF-07293623 (100 nM, 6 hours, n=3). (**D**) ChIP-qPCR analysis of AR binding at target genes in LuCaP 176 cells. Fold enrichment over normal IgG is shown at *FKBP5*, *KLK3*, and *TMPRSS2* loci after 72 h treatment with DMSO or PF-07293623 (100 nM for 72 h, n=3). (**E**) GSEA of CP reactome (MSigDB) for the 465 upregulated proteins identified by HPG-TMT mass spectrometry upon PF-07293623 treatment. Dot size indicates gene ratio; color reflects $-\log_{10}$ p-value. (**F**) GSEA of CP reactome (MSigDB) for the top 500 downregulated proteins in the HPG-TMT upon PF-07293623 (100 nM, 6 h). Dot size indicates gene ratio; color reflects $-\log_{10}$ p-value. (**G**) Schematic of HPG-TMT proteome profiling (left) and genome-wide CRISPRi AR-GFP reporter screen (right) used to identify positive regulators of AR protein expression. Intersection yielded 36 candidates as positive regulators of AR expression. (**H**) Volcano plot of genome-wide CRISPRi screen with AR-GFP reporter cells showing AR, as a positive control. Significant gene hits (FDR < 0.05) are shown in purple. (**I**) mRNA transcript levels of *BAP1*, *GOLGA5*, *OTUD3*, *UBA2*, *VBP1* in DMSO or PF-07293623 (100 nM, 72 hours) treated LuCaP 176 cells. (**J**) Representative immunoblot (left) and quantification (right) of candidate AR regulators (GOLGA5, UBA2, and VBP1) in LuCaP 176 cells (left) treated with DMSO vs PF-07293623 (100 nM, 72 hours). All plots represent mean ± SEM. Statistical significance was determined using a two-tailed t-test. n.s. = not significant ($p > 0.05$); *$p < 0.05$; **$p < 0.01$.

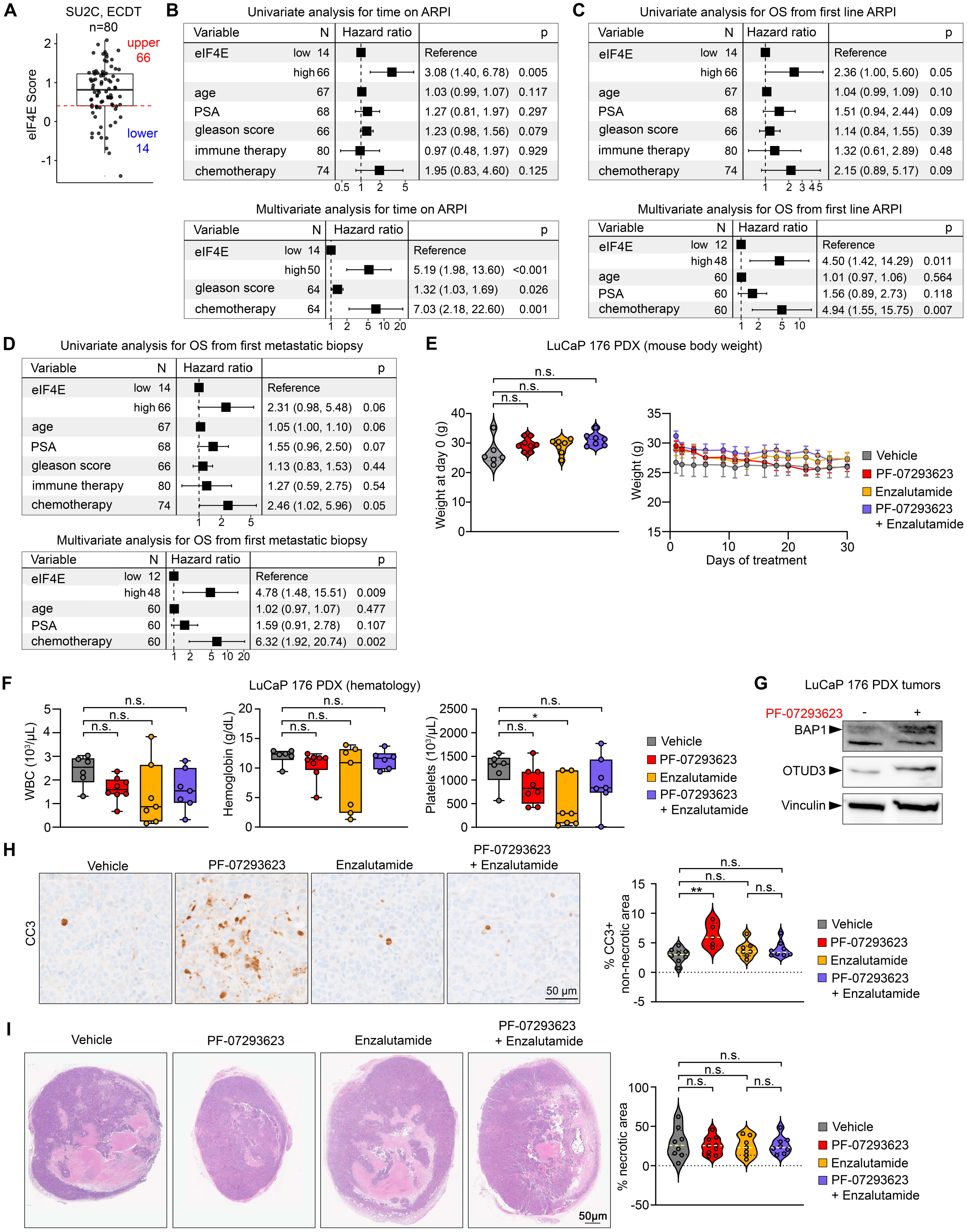
A
SU2C, ECDT
n=80
eIF4E Score
upper 66
lower 14
B
Univariate analysis for time on ARPI
Variable | N | Hazard ratio | p
eIF4E low 14 Reference
high 66 3.08 (1.40, 6.78) 0.005
age 67 1.03 (0.99, 1.07) 0.117
PSA 68 1.27 (0.81, 1.97) 0.297
gleason score 66 1.23 (0.98, 1.56) 0.079
immune therapy 80 0.97 (0.48, 1.97) 0.929
chemotherapy 74 1.95 (0.83, 4.60) 0.125
Multivariate analysis for time on ARPI
eIF4E low 14 Reference
high 50 5.19 (1.98, 13.60) <0.001
gleason score 64 1.32 (1.03, 1.69) 0.026
chemotherapy 64 7.03 (2.18, 22.60) 0.001
C
Univariate analysis for OS from first line ARPI
eIF4E low 14 Reference
high 66 2.36 (1.00, 5.60) 0.05
age 67 1.04 (0.99, 1.09) 0.10
PSA 68 1.51 (0.94, 2.44) 0.09
gleason score 66 1.14 (0.84, 1.55) 0.39
immune therapy 80 1.32 (0.61, 2.89) 0.48
chemotherapy 74 2.15 (0.89, 5.17) 0.09
Multivariate analysis for OS from first line ARPI
eIF4E low 12 Reference
high 48 4.50 (1.42, 14.29) 0.011
age 60 1.01 (0.97, 1.06) 0.564
PSA 60 1.56 (0.89, 2.73) 0.118
chemotherapy 60 4.94 (1.55, 15.75) 0.007
D
Univariate analysis for OS from first metastatic biopsy
eIF4E low 14 Reference
high 66 2.31 (0.98, 5.48) 0.06
age 67 1.05 (1.00, 1.10) 0.06
PSA 68 1.55 (0.96, 2.50) 0.07
gleason score 66 1.13 (0.83, 1.53) 0.44
immune therapy 80 1.27 (0.59, 2.75) 0.54
chemotherapy 74 2.46 (1.02, 5.96) 0.05
Multivariate analysis for OS from first metastatic biopsy
eIF4E low 12 Reference
high 48 4.78 (1.48, 15.51) 0.009
age 60 1.02 (0.97, 1.07) 0.477
PSA 60 1.59 (0.91, 2.78) 0.107
chemotherapy 60 6.32 (1.92, 20.74) 0.002
E
LuCaP 176 PDX (mouse body weight)
Weight at day 0 (g)
Weight (g)
Days of treatment
n.s.
Vehicle
PF-07293623
Enzalutamide
PF-07293623 + Enzalutamide
F
LuCaP 176 PDX (hematology)
WBC (10³/μL)
Hemoglobin (g/dL)
Platelets (10³/μL)
n.s.
*
G
LuCaP 176 PDX tumors
PF-07293623 - +
BAP1
OTUD3
Vinculin
H
Vehicle
PF-07293623
Enzalutamide
PF-07293623 + Enzalutamide
CC3
50 μm
% CC3+ non-necrotic area
**
n.s.
I
% necrotic area
50μm

Supplemental Figure S7. Effect of eIF4E cap-binding domain inhibition and enzalutamide combination treatment in LuCaP PDX models.

(**A**) Patients were categorized based on eIF4E expression into eIF4E-low (below the 25th percentile, n = 14) and eIF4E-high (above the 25th percentile, n = 66) groups. (**B**) Univariate and multivariate analysis of clinical features associated with time on ARPI treatment in SU2C cohort. (**C**) Univariate and multivariate analysis of clinical features associated with OS from start of ARPI treatment in SU2C cohort. (**D**) Univariate and multivariate analysis of clinical features associated with OS from first biopsy in SU2C cohort. (**E**) Mouse weights of LuCaP 176 PDX at start of the trial (left) and over the 30-day treatment period (right) across various treatment groups: vehicle, PF-07293623, enzalutamide and the combination of PF-07293623 and enzalutamide, n=6 or more mice per group. (**F**) White blood cell, hemoglobin and platelet count of LuCaP 176 PDX mice on trial. (**G**) Representative immunoblot of BAP1 and OTUD3 levels in LuCaP 176 PDX tumors upon vehicle and PF 07293623 treatment. (**H**) Cleaved caspase-3 (CC3) immunohistochemical staining in LuCaP 176 PDX tumor sections (left, scale bar = 50 µm). Graph (right) shows quantification of the percentage of CC3-positive staining in non-necrotic areas. (**I**) Hematoxylin and eosin (H&E) staining of LuCaP 176 PDX tumor sections (right, scale bar represents 50 µm). Graph (right) shows quantification of the percentage of necrotic area. All plots represent mean ± SEM. Significance was determined by one way ANOVA with Sidak's multiple comparisons test in **E,F,H,I**. n.s. = not significant ($p > 0.05$); *$p < 0.05$; **$p < 0.01$.

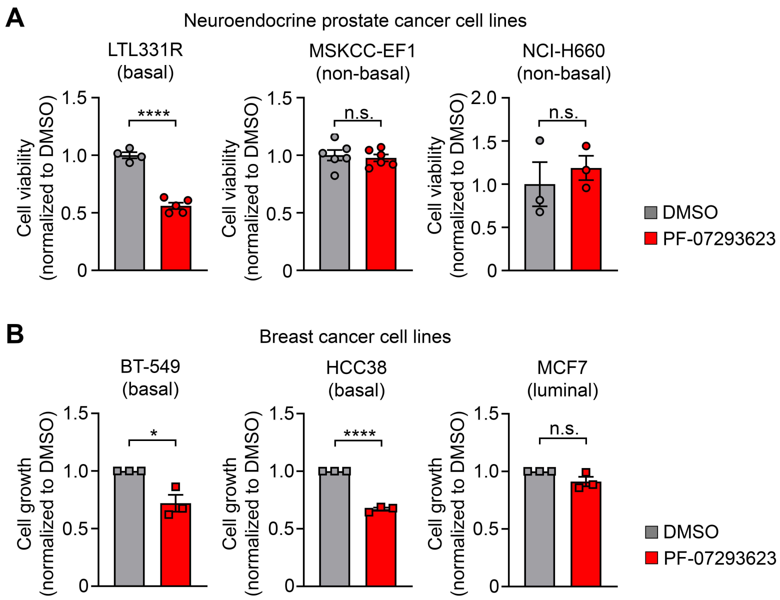
A
Neuroendocrine prostate cancer cell lines
LTL331R
(basal)
MSKCC-EF1
(non-basal)
NCI-H660
(non-basal)
Cell viability
(normalized to DMSO)
****
n.s.
n.s.
DMSO
PF-07293623
B
Breast cancer cell lines
BT-549
(basal)
HCC38
(basal)
MCF7
(luminal)
Cell growth
(normalized to DMSO)
*
****
n.s.
DMSO
PF-07293623

Supplemental Figure S8. Basal phenotype cancers are sensitive to eIF4E cap-binding domain inhibition.

(**A**) Fold change in cell viability of NEPC cell lines treated with PF-07293623, comparing basal phenotype (LTL331R) with non-basal phenotype (MSKCC-EF1 and NCI-H660) cells, measured by CellTiter-Glo and normalized to DMSO control. (**B**) Fold change in cell growth of breast cancer cell lines treated with PF-07293623, comparing basal phenotype (BT549 and HCC38) with luminal phenotype (MCF7) cells, normalized to DMSO control. All plots represent mean ± SEM. Statistical significance was determined using a two-tailed t-test. n.s. = not significant ($p > 0.05$); *$p < 0.05$; ****$p < 0.0001$.

Supplemental Tables

Supplemental Table 1. HPG-TMT mass spectrometry of LuCaP 176 cells treated with and without PF-07293623 for 6 hours.

Supplemental Table 2. RNA-seq of of LuCaP 176 cells treated with and without PF-07293623 for 6 hours.

Supplemental Table 3. List of Genes with AGCCWCCAGC motif in 5′ UTRs (FIMO Analysis) of top HPG-TMT mass spectrometry candidates of LuCaP 176 cells treated with and without PF-07293623 for 6 hours.

Supplemental Table 4. List of cell Lines and mouse models.

Supplemental Table 5. List of primary antibodies.

Supplemental Table 6. List of qPCR primers.

Supplemental methods

AR immunofluorescence

Cells on chamber slides (Lab-Tek, cat. #154941) were fixed with 4% PFA for 10 minutes, permeabilized with 0.1% Triton X-100 in PBS for 10 minutes and blocked with 1% BSA and 5% Goat Serum solution for 2 hours. Anti-AR primary antibody (Supplemental Table 5) was applied overnight at 4 °C, followed by Alexa Fluor 594 (Invitrogen, cat. #A11037) secondary for 1 hour at RT. Slides were mounted using Pro-Long gold with DAPI (Fisher Scientific, cat. #P36935), imaged using an Aperio Scanscope FL (Leica) and analyzed for AR and DAPI co-localization using ImageJ.

IHC and H&E Staining

Mice xenograft tumors were harvested on day 30, washed with PBS, fixed in 10% neutral-buffered formalin (Thermo Fisher, cat. #22-050-104) at 4 °C overnight, embedded in paraffin and sectioned. For AR IHC, DISCOVERY XT platform (Ventana Medical Systems, Inc., Tucson, U.S.A) was used with the Discovery DAB Map Detection Kit. For CC3 IHC, slides were baked, deparaffinized and antigen retrieval was performed in citric acid (Vector Labs, cat. #H-3300, pH 6.0) at 120 °C for 30 minutes. Endogenous peroxidase activity and nonspecific binding were blocked, followed by overnight primary antibody (Supplemental Table 5) incubation at 4 °C. Slides were incubated with anti-rabbit or anti-mouse HRP for 1 hour, detected with DAB (Dako, cat. #K3467), counterstained with hematoxylin (Agilent, cat. #S3309), mounted (Dako, cat. #S3025) and scanned using Ventana DP200 (Roche). Images were analyzed using HALO (Indica Labs, v.3.6) with tissue classifiers trained to distinguish stroma, necrosis and tumor. Analysis was restricted to non-necrotic regions with a 50 µm margin. The Cytonuclear module quantified cell counts and marker positivity, classifying AR and pH3 staining (none, weak, moderate, or high) with moderate/high staining compared to controls. Six or more ROIs per tumor were analyzed.
