# Supplementary material for: Therapeutic targeting of the eIF4E cap-binding domain reveals control of lineage fate in prostate cancer
Source: J Clin Invest. 2026 Apr 14;136(12):e199838. doi: 10.1172/JCI199838 (PMC13262746; doi:10.1172/JCI199838)

Full unedited western blots for Figure 2D.

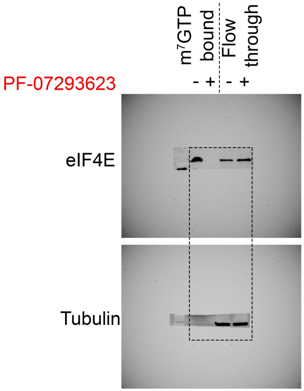

Full unedited western blots for Figure 2E.

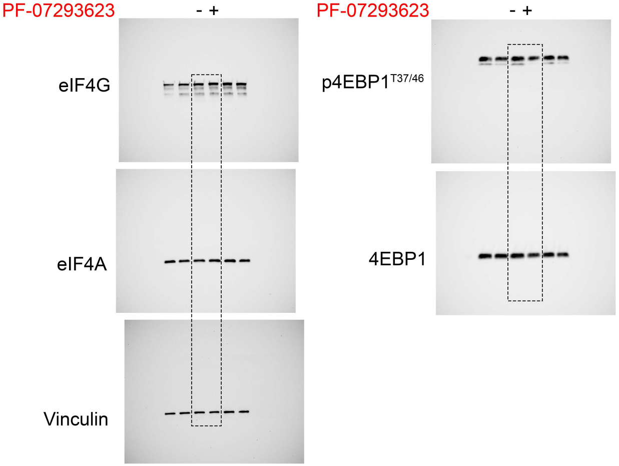

Full unedited western blots for Figure 2F.

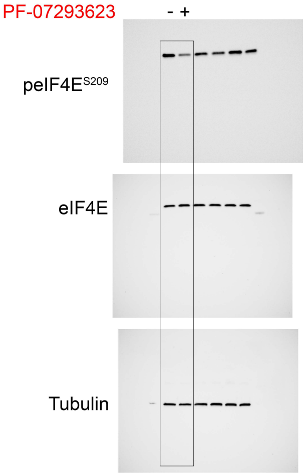

Full unedited western blots for Figure 2G.

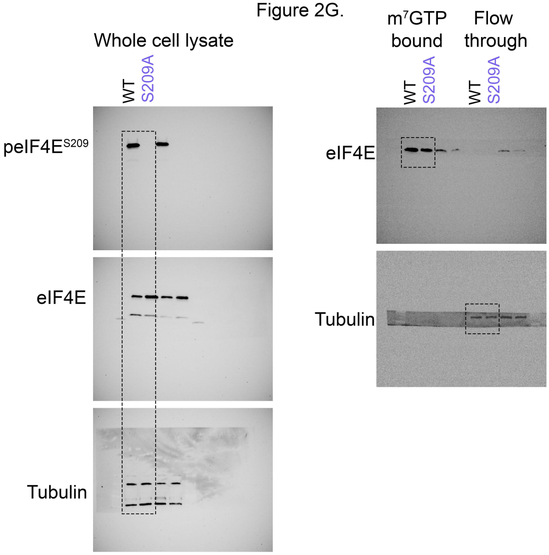

Full unedited western blots for Figure 2D.

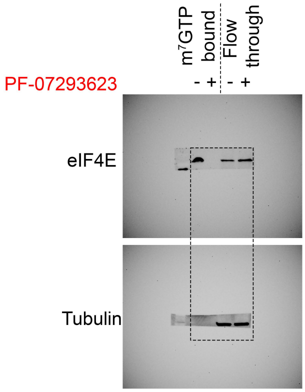

Full unedited western blots for Figure 2E.

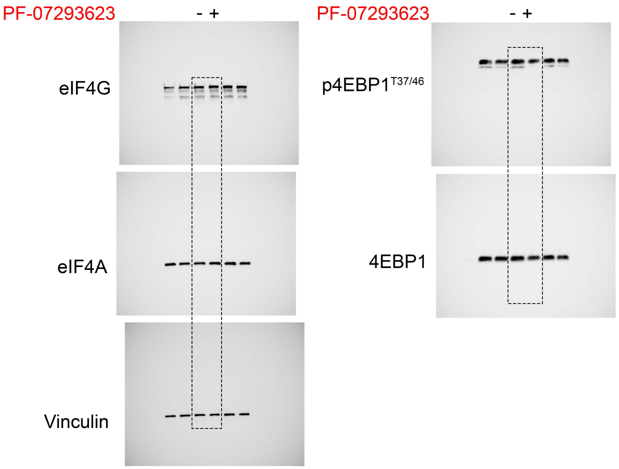

Full unedited western blots for Figure 2F.

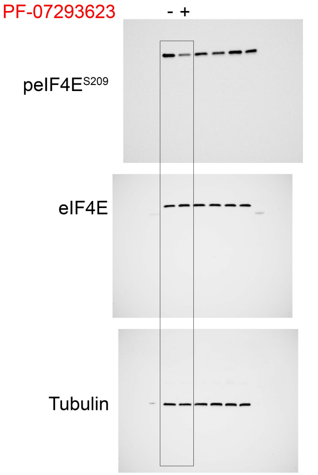

Full unedited western blots for Figure 2G.

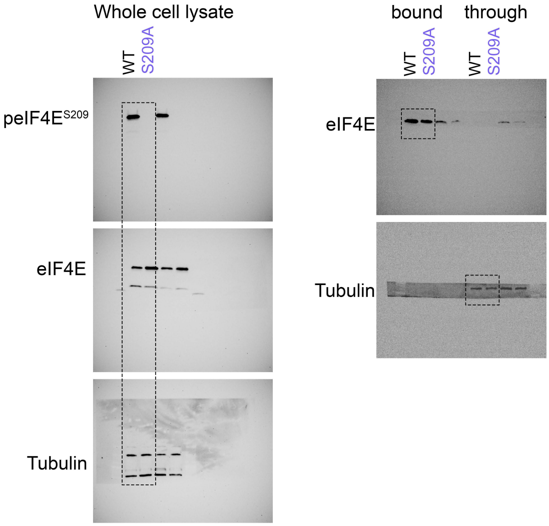

Full unedited western blots for
Figure 3F.

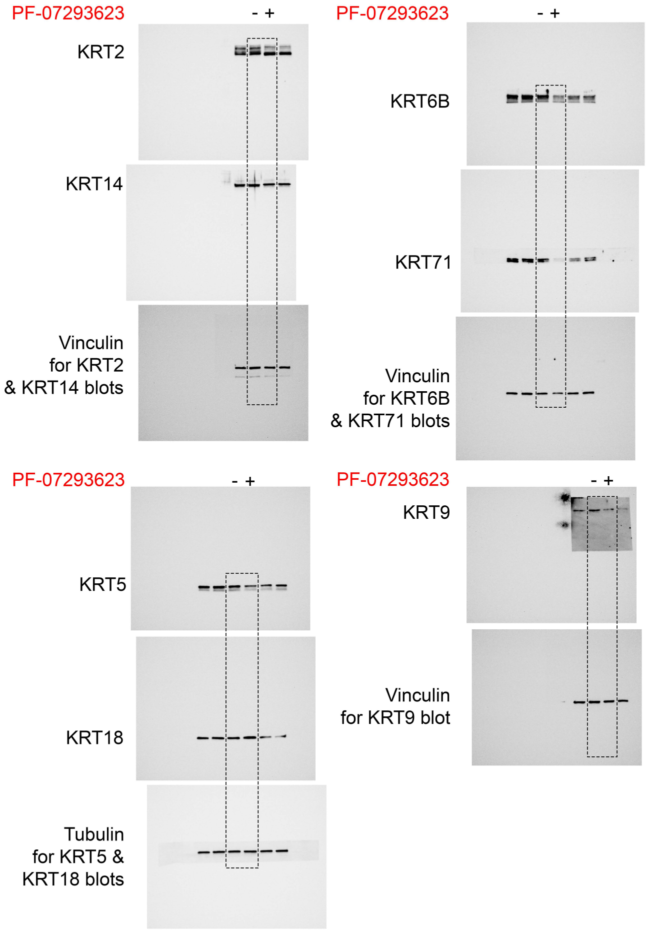

Full unedited western blots for
Figure 4F.

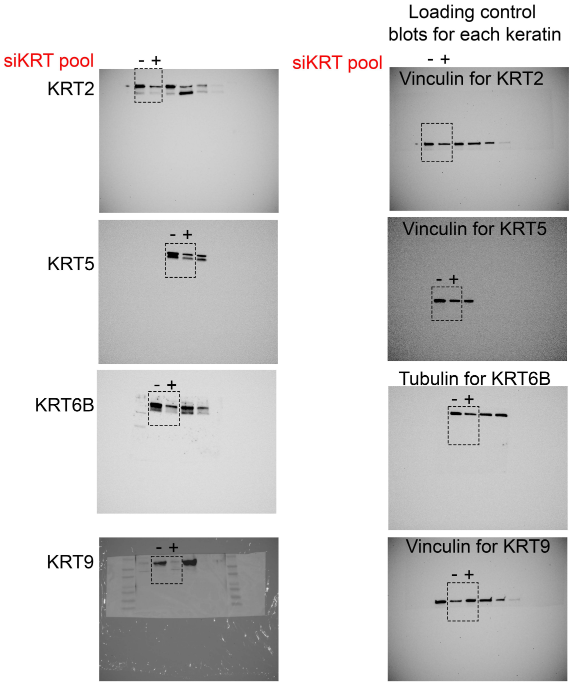

siKRT pool :siKRT2 + siKRT5 + siKRT6B + siKRT9

Full unedited western blots for
Figure 4H.

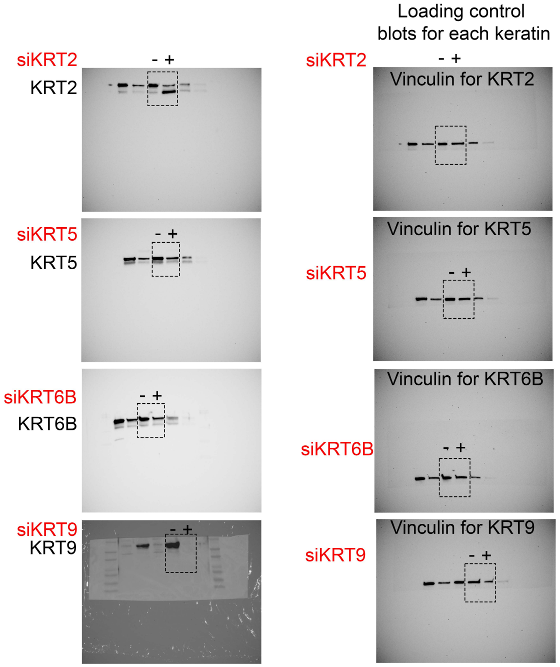

Full unedited western blots for Figure 5G.

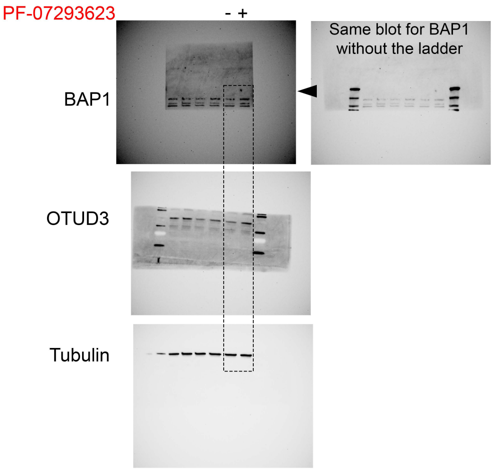

Full unedited western blots for Figure 5H.

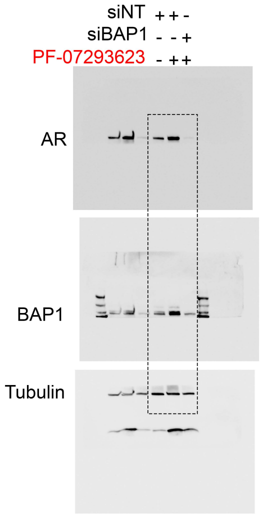

Full unedited western blots for Figure 5I.

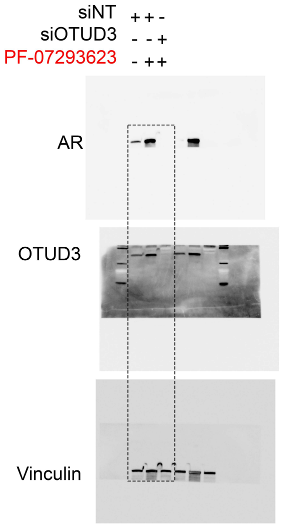

Full unedited western blots for Figure 5J.

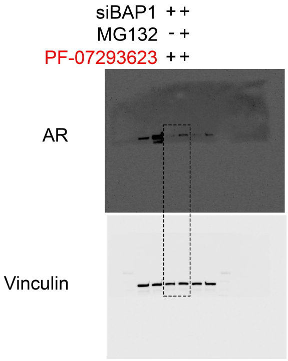

Full unedited western blots for Figure 5K.

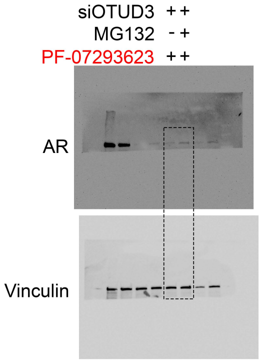

Full unedited western blots for Supplemental Fig 6J.

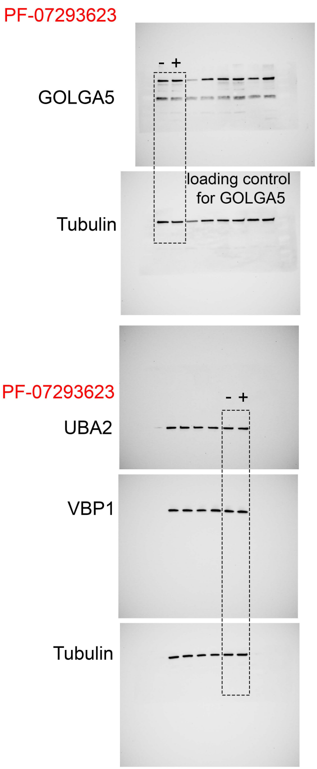

Full unedited western blots for Supplemental Fig 7G.

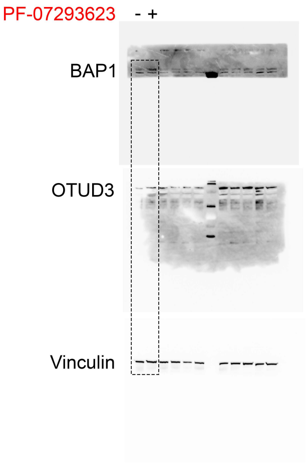

Supplement: Unedited blot and gel images [file jci-136-199838-s108.pdf]
